# Initiation: A Critical Step for High Activity and Stability in Ni‐Based Methane Dry Reforming Catalysts Supported on *θ*‐Al_2_O_3_

**DOI:** 10.1002/anie.1716058

**Published:** 2026-03-25

**Authors:** Wei Wang, Milivoj Plodinec, Wei Zhou, Christophe Copéret

**Affiliations:** ^1^ Department of Chemistry and Applied Biosciences ETH Zürich Zürich Switzerland; ^2^ ScopeM ETH Zürich Zürich Switzerland

**Keywords:** carbide cycle, dry reforming of methane, induction period, Ni‐based catalyst

## Abstract

Ni‐based catalysts are extensively studied for the dry reforming of methane (DRM), which converts CO_2_ and CH_4_—the two most abundant greenhouse gases—into syngas for downstream chemical synthesis. The harsh reaction conditions required for DRM lead to coking, metal aggregation. Although multiple mechanisms have been proposed, the molecular‐level understanding of the reaction remains debated. Here, we report the synthesis of *θ*‐Al_2_O_3_‐supported Ni DRM catalysts via surface organometallic chemistry (SOMC) and report its outstanding activity and stability. The resulting Ni nanoparticles remain highly dispersed, with an average size of 5.3 ± 1.3 nm even after reduction at 900°C. This model catalyst exhibits distinct temperature‐dependent behavior during DRM, with marked structural and mechanistic differences observed within a narrow 50°C range. In situ x‐ray absorption spectroscopy (XAS) and ex situ synchrotron x‐ray diffraction (XRD) reveal a dynamic induction process involving rapid Ni oxidation, followed by reduction and carbon insertion into the Ni lattice at 850°C, forming a carbide‐like NiC_x_ phase. At 800°C, incorporation of carbon is limited, thus leading to surface coking and catalyst deactivation. Furthermore, gas‐switching experiments confirm the importance of a carbide cycle at 850°C, enabling continuous carbon removal and sustained catalytic stability.

## Graphical abstract

A well‐defined Ni/*θ*‐Al_2_O_3_ catalyst prepared by surface organometallic chemistry exhibits sharp temperature‐dependent behavior in dry reforming of methane. At 850°C, a dynamic induction process forms a transient carbide‐like NiC_x_ phase that enables continuous carbon removal and coke resistance, whereas at 800°C incomplete carbon insertion leads to filamentous coke formation and catalyst deactivation.

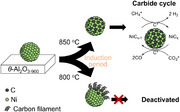

## 1 Introduction

Emissions of greenhouse gases (GHGs), for example CO_2_ and CH_4_, are a major cause of global environmental challenges such as climate change, ocean acidification, sea level rise, and extreme weather events [1, 2, 3]. In this context, dry reforming of methane (DRM) offers a strategy to simultaneously convert CO_2_ and CH_4_ into syngas (a mixture of CO and H_2_), a versatile feedstock for producing value‐added chemicals through established technologies, for example methanol synthesis or Fischer‐Tropsch (FT) process [4, 5, 6]. Although solar‐driven photothermal DRM has emerged as a promising and sustainable approach that uses solar energy to drive the reaction, with recent studies indicating that solar irradiation can modulate the electronic structure, activate lattice oxygen, and enhance oxygen migration [7, 8], it remains far from industrial application when compared with conventional thermal DRM. Ni‐based catalysts have attracted extensive attention for thermal DRM owing to their high activity and low cost. Several reaction mechanisms have been considered involving redox, vacancy, and carbide cycles, considering the facilitation of CO_2_ activation and the prevention of carbon deposition on active sites [3, 9, 10, 11, 12, 13, 14, 15, 16, 17, 18, 19, 20, 21, 22, 23]. However, the complexity of the catalyst structures can significantly impede mechanistic studies, hindering the establishment of reliable structure–performance relationships [24, 25, 26, 27, 28, 29]. Moreover, a major limitation of these catalysts also lies in the rather rapid deactivation, caused by carbon deposition and Ni nanoparticle sintering. In addition, *γ*‐Al_2_O_3_, a widely employed support due to its high surface area and mechanical stability, suffers from the presence of intrinsic defects that often promote the formation of bulk spinel phases with Ni, ultimately leading to loss of active Ni‐sites and deactivation [21]. In contrast, *θ*‐Al_2_O_3_ is thermally more stable and exhibits a lower density of surface defects while retaining comparable mechanical stability [30]. Consequently, *θ*‐Al_2_O_3_ has been employed to mitigate the deleterious processes discussed above [2, 4, 5, 6].

In this regard, surface organometallic chemistry (SOMC) has emerged as a powerful strategy for constructing tailored supported nanoparticles and model catalysts, with controlled compositions and interfaces [24, 25, 26, 31]. When combined with advanced operando spectroscopic techniques, this approach provides direct insight into catalyst structural evolution under working conditions and facilitates the establishment of structure–performance relationships [31]. In this work, we thus employ SOMC approach to form narrowly distributed small (ca. 5 nm) Ni nanoparticles dispersed on *θ*‐Al_2_O_3_, an alumina phase that displays superior thermal and hydrothermal stability, while maintaining a high surface area at elevated temperatures [30, 32]. The resulting Ni‐based catalyst exhibits high activity and stability at 850°C, exhibiting a pronounced temperature dependence within a narrow temperature window. Notably, the induction period observed at the onset of reaction is found to play a pivotal role in governing the enhanced activity and stability. In situ x‐ray absorption spectroscopy (XAS) enables to track the dynamic evolutions of the catalyst under DRM conditions including the critical activation period. X‐ray diffraction (XRD) analysis and hydrogen temperature‐programmed reduction (H_2_‐TPR) indicate carbon insertion into the Ni lattice; this carbide‐like phase, formed during the induction period at higher reaction temperature (850°C), appears to mitigate carbon accumulation and to improve catalyst stability. In sharp contrast, at lower temperature (800°C), carbon readily deposits on the Ni surface, blocking active sites and ultimately leading to catalyst deactivation.

## 2 Results and Discussion

### 2.1 Preparation and Characterization of Supported Nickel Nanoparticles

**FIGURE 1:**
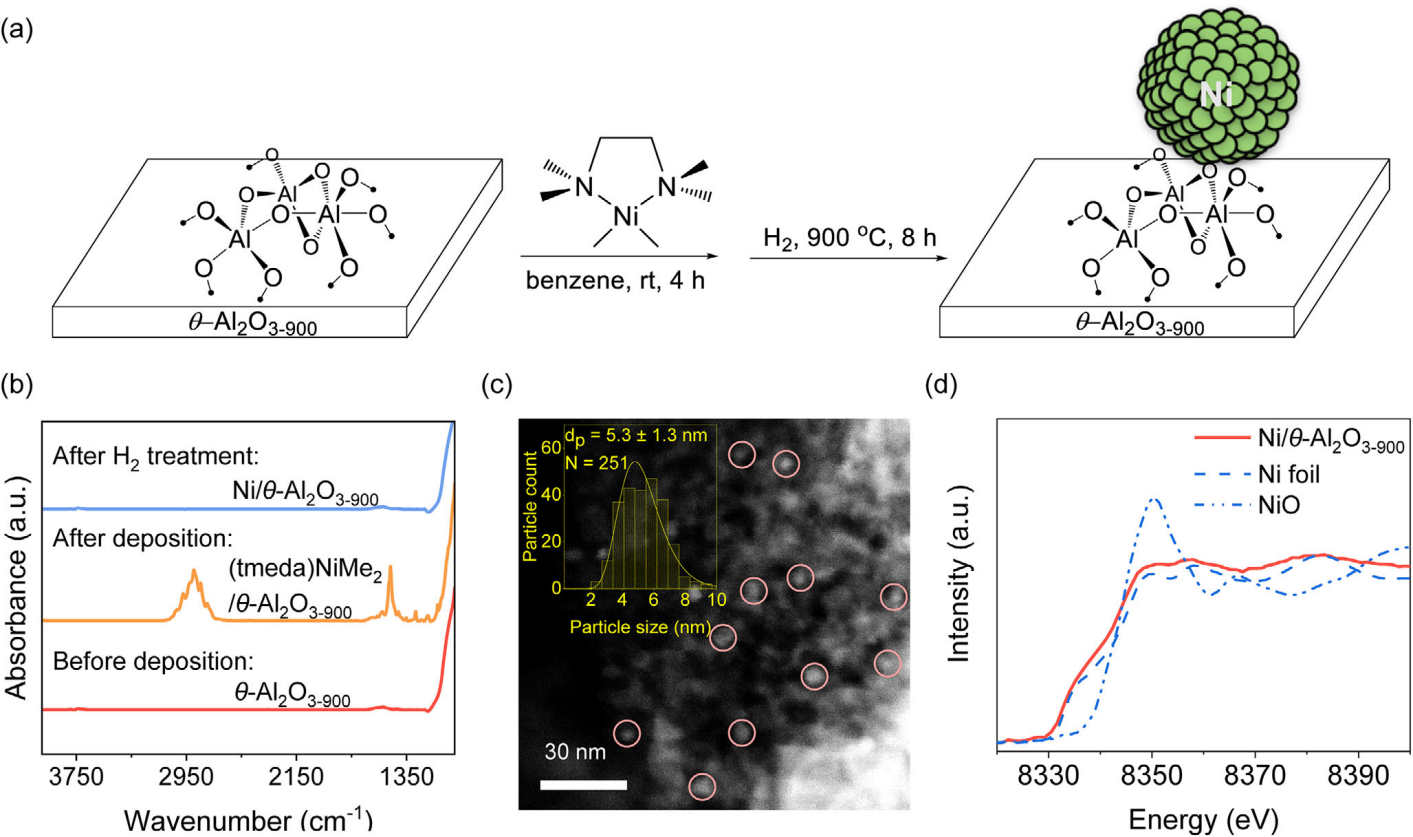
Preparation of Ni/*θ*‐Al_2_O_3‐900_ material. (a) The illustration of the synthetic approach for Ni/*θ*‐Al_2_O_3‐900_ via SOMC approach. (b) The FTIR spectra before deposition, after deposition, and after H_2_ treatment during the synthesis process. (c) Representative high‐angle annular dark‐field STEM (HAADF‐STEM) image of Ni nanoparticles (highlighted by pink circles) and corresponding particle size distribution (PDS) (inset) of Ni/*θ*‐Al_2_O_3‐900_ catalyst. (d) In situ XANES spectrum at the Ni K‐edge under hydrogen flow (850°C, 1 bar) of the fresh catalyst.

*θ*‐Al_2_O_3‐900_‐supported Ni nanoparticles (Ni/*θ*‐Al_2_O_3‐900_) are first synthesized via SOMC approach (Figure 1a; see Supporting Information for experimental details). First, the *θ*‐Al_2_O_3_ support is synthesized via staged calcination of bayerite, according to a reported procedure [32], and subsequently treated under high vacuum at 900°C to yield *θ*‐Al_2_O_3‐900_ (surface area = 148 m^2^·g^−1^ and OH density = 0.06 –OH·nm^−2^ as determined by titration, see Figure S1). We next deposit Ni using tetramethylethylenediamine(dimethyl)nickel(II) [(tmeda)NiMe_2_] as a molecular precursor, that grafts onto *θ*‐Al_2_O_3‐900_ (nominal loading 1.1 wt% Ni, surface density 0.8 Ni·nm^−2^). All synthetic steps are monitored by Transmission IR spectroscopy (Figure 1b). Prior to deposition, the IR spectrum exhibits a minor –OH stretching band consistent with the titrated –OH density (Figure S1). Despite the negligible –OH density on the *θ*‐Al_2_O_3‐900_ surface, over 91% of (tmeda)NiMe_2_ is grafted, as determined by ^1^H NMR analysis of the residual precursor in the supernatant (Figure S2), suggesting that the precursor interacts with surface Al–O–Al and residual Al–OH sites after grafting. This grafting process is further corroborated by IR spectroscopy, which shows the disappearance of Al–OH vibrational bands (3650–3800 cm^−1^) and the appearance of C─H stretching vibrational bands (2800–3000 cm^−1^), C─H bending vibrational bands (1250–1640 cm^−1^), and a C─N stretching band at 1460 cm^−1^ (Figures 1b and S3), characteristic of the organic ligands of the Ni precursor [33]. Following subsequent H_2_ treatment, these features disappear completely, indicating full removal of the ligands and formation of a clean, ligand‐free material, with restoration of the weak –OH band. The reduction treatment at 900°C yields small Ni nanoparticles with a narrow size distribution (5.3 ± 1.3 nm, Figure 1c) as determined by scanning transmission electron microscopy (STEM). This Ni nanoparticles obtained by SOMC is smaller and more homogeneous than these of Ni‐based catalysts prepared by conventional method such as impregnation [34]. Inductively coupled plasma optical emission spectroscopy (ICP‐OES) analysis indicates a nickel loading of 1.05 wt%.

The XRD pattern of the fresh catalyst displays weak Ni reflections, aside from minor intensity variations from the support, likely arising from the small crystallite size of the Ni nanoparticles in this material (Figure S4a). The XANES spectrum of fresh Ni/*θ*‐Al_2_O_3‐900_ (Figure 1d) closely resembles that of the Ni foil reference, with coincident pre‐edge (8333 eV) and edge (8345 eV) positions [21]. Linear combination fitting (LCF) of the XANES data using the Ni foil and NiO as references (Figure S5a) indicates that 90% of the nickel species are reduced to Ni^0^ under H_2_ flow at 850°C. The extended x‐ray absorption fine structure (EXAFS) analysis of the pristine Ni/*θ*‐Al_2_O_3‐900_ sample is satisfactorily modeled using both Ni–Ni and Ni–O scattering paths. The best fit yields an average coordination number (CN) of 10.5 for Ni–Ni and 0.3 for Ni–O (Figure S6 and Table S2), suggesting that the majority of Ni atoms reside in a metallic environment, while only a minor fraction remains coordinated to surface oxygen species, possibly interfacial sites, which is characteristic of small, well‐defined nanoparticles prepared by the SOMC approach.

### 2.2 DRM Evaluation

**FIGURE 2:**
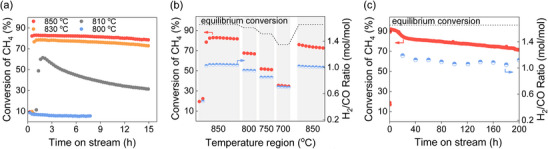
DRM performance of the Ni/*θ*‐Al_2_O_3‐900_ catalyst tested at different temperatures. (a) CH_4_ conversion during DRM tests under different temperatures. (b) CH_4_ conversion and H_2_/CO mol ratio when changing the temperature after the induction at 850°C. Reaction condition in (a) and (b): *F* = 50 mL·min^−1^ of CH_4_/CO_2_/N_2_ (2/2/1), 20 mg of catalyst, GHSV = 150 000 mL·h^−1^·g_cat_^−1^, P = 1 bar. (c) The Ni/*θ*‐Al_2_O_3‐900_ catalyst stability test. Reaction condition: *F* = 20 mL·min^−1^ of CH_4_/CO_2_/N_2_ (2/2/1), 20 mg of catalyst, GHSV = 60 000 mL·h^−1^·g_cat_^−1^, P = 1 bar.

Next, the catalytic performance of Ni/*θ*‐Al_2_O_3‐900_ is evaluated in DRM with a flow of 50 mL min^−1^ of CH_4_/CO_2_/N_2_ (2:2:1) at 1 bar at a given temperature. Prior to the reaction, the catalyst is reduced under H_2_ (1 bar, 1 h) at 870°C. The Ni/*θ*‐Al_2_O_3‐900_ shows low CH_4_ conversion (6%–9%) at 800°C (Figure 2a). Upon increasing the temperature to 810°C, CH_4_ conversion improves significantly albeit with severe deactivation after reaching the highest conversion of ca. 62%. Further increasing the temperature to 830°C and 850°C leads to a further increase in conversion, 78% and 81%, respectively, while also increasing the stability as illustrated by the stable conversion for at least 15 h. The Ni/*θ*‐Al_2_O_3‐900_ catalyst achieves a CH_4_ consumption rate of 60.2 mmol·g_Ni_^−1^·s^−1^ at 850°C, outperforming most previously reported Ni‐based catalysts (Table S1). Note that the CO_2_ conversion closely matches the CH_4_ conversion within the temperature range of 800°C–850°C, which is consistent with the expected reaction stoichiometry for DRM (Figure S9).

Notably, an induction period of ca. 60 min is observed at 850°C before DRM reaches its steady‐state activity. To elucidate the role of the induction period, the catalyst is first stabilized at 850°C, after which the reaction temperature is decreased stepwise to 700°C (Figure 2b). Of interest, the CH_4_ conversion remains at ∼70% upon cooling to 800°C (Figure 2b), substantially higher than when the reaction is initiated directly at 800°C (Figure 2a). This suggests that a distinct, more active structure forms during the high‐temperature induction period, despite relatively small difference (50°C) compared to initial temperature. Remarkably, the catalyst retains high activity even at 700°C and almost recovers full performance upon returning to 850°C (Figure 2b).

Note that under such reaction conditions, DRM is usually accompanied by reverse water‐gas shift (RWGS) reaction as a competing side reaction, consuming H_2_ and lowering the H_2_/CO ratio below the stoichiometric value of 1.0 [19, 35, 36]. It is observed that the H_2_/CO ratio increases with increasing reaction temperature over Ni/*θ*‐Al_2_O_3‐900_, reaching a value of 1.0 at 850°C (Figure 2b). These observations are consistent with thermodynamic predictions: higher temperatures are less favorable for RWGS [5, 37]. For the long‐term stability test, a relatively low GHSV was employed to minimize cold spots due to the highly endothermic DRM process [38]. During the initial 30 h of the stability test, methane conversion decreases rapidly due to competition between carbon insertion and carbon accumulation on the Ni surface, where net carbon buildup blocks active sites, until a dynamic equilibrium is established and the catalytic activity stabilizes. Eventually, Ni/*θ*‐Al_2_O_3‐900_ maintains over 80% of the initial CH_4_ conversion and a nearly constant H_2_/CO ratio at around 1.0 throughout a 200 h stability test at 850°C (Figure 2c).

### 2.3 Investigation of the Induction Period

**FIGURE 3:**
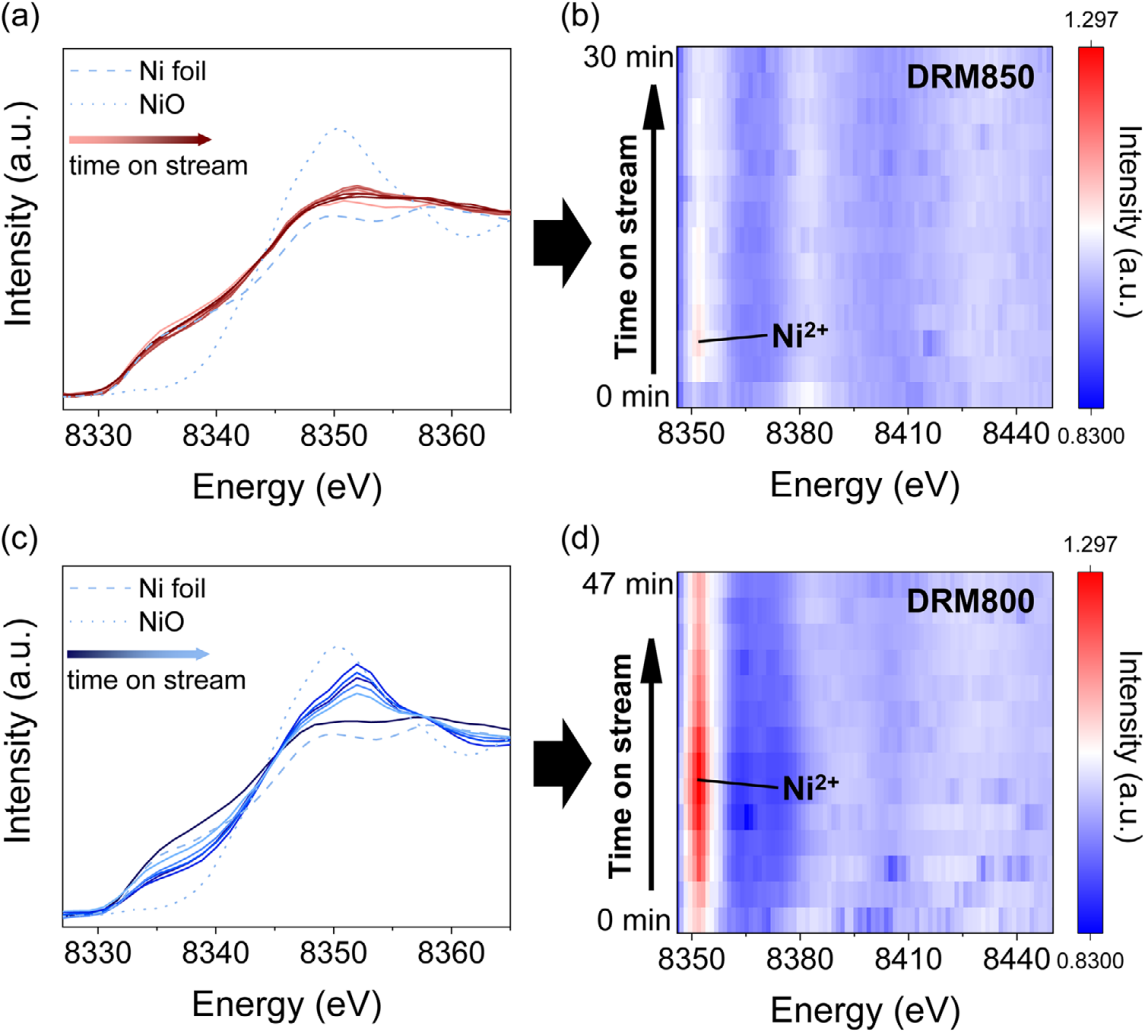
In situ XAS studies at the Ni K‐edge: Dynamic evolution of the Ni K‐edge XANES spectra under 850°C (a) and 800°C (c), with corresponding heat maps of spectral intensity (b and d) collected during the induction period.

To gain insight into the effect of different initiation reaction temperatures and catalyst evolution during the induction period, we next carry out a series of in situ XAS experiments. Ni K‐edge XANES spectra are collected during DRM at 850°C (DRM850) and 800°C (DRM800), respectively. At the onset of DRM850, both the white line intensity and the edge position increase stepwise during the initial phase of the reaction, indicating progressive Ni oxidation (Figure 3a,b). Subsequently, the white line intensity decreases and the edge position gradually shifts back to lower energy over time on stream, suggesting reduction of the oxidized Ni species. A similar trend is observed during DRM800 (Figure 3c,d); however, a larger shift to higher energy on the edge position and higher white line intensity are observed after the induction period, paralleling with the rapid deactivation of the catalyst during the DRM800 test (Figure 2a). LCF analysis of the Ni K‐edge XANES spectra reveals that at the onset of DRM850, up to 29% of Ni is oxidized to Ni^2+^, which is significantly lower than under DRM800 conditions (65%) (Figure S10). Furthermore, after the induction period, a higher fraction of Ni^0^ is retained at 850°C (82%) than at 800°C (71%) (Figure S10). These results demonstrate that gas mixture reaction at 850°C minimizes over‐oxidation and favors preservation of metallic Ni, which is likely critical for sustaining high DRM activity.

### 2.4 Investigation of the Spent Catalysts

**FIGURE 4:**
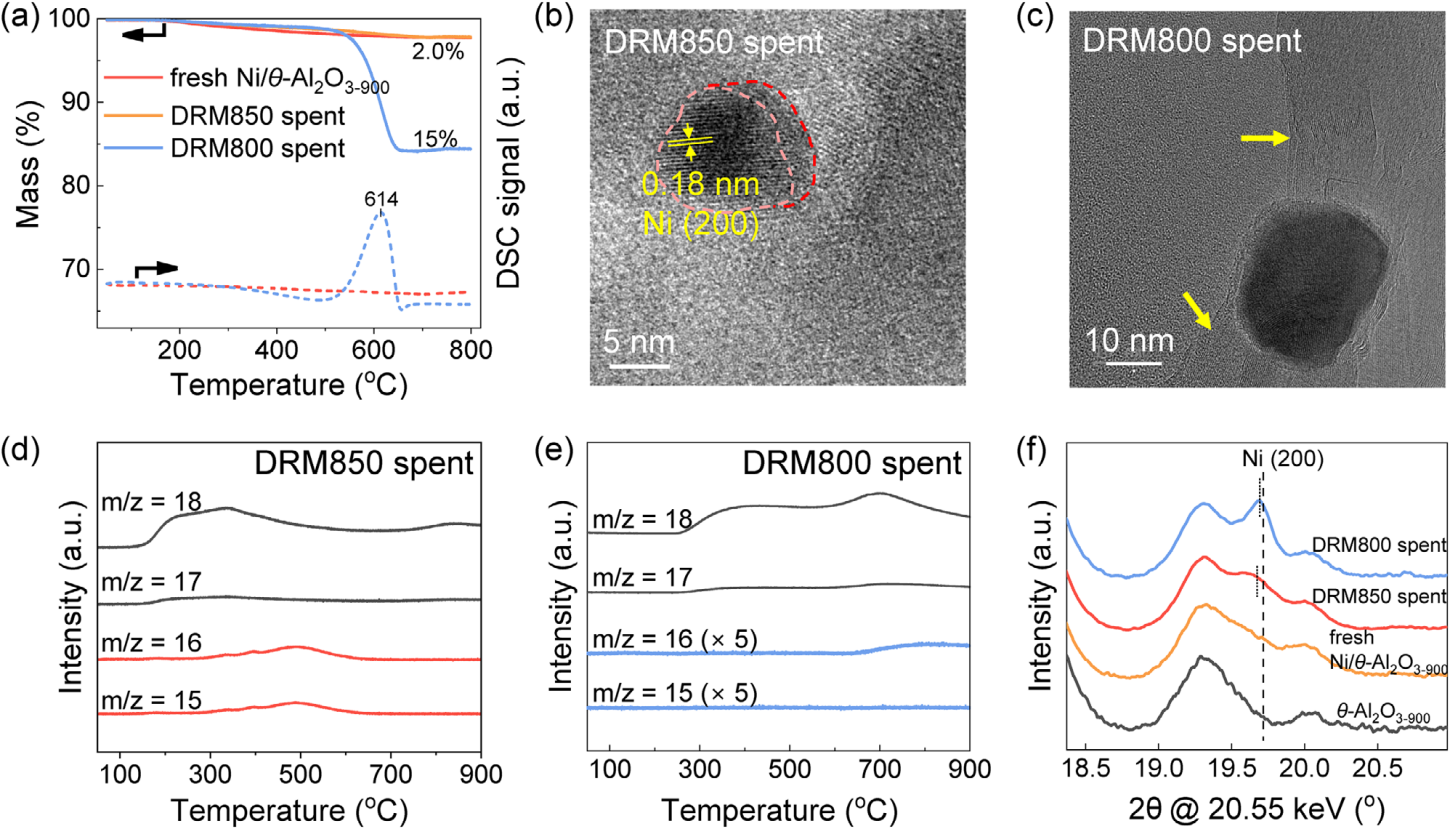
Characterizations for the spent catalysts (DRM850 spent, and DRM800 spent): TGA and DSC profiles of the spent samples (a). High‐resolution TEM (HRTEM) image showing lattice fringes of a Ni nanoparticle in the DRM850 spent sample (the crystalline region is outlined by a pink dashed line, and the nanoparticle–support interface is marked by a red dashed line) (b). HRTEM image highlighting carbon nanotubes on DRM800 spent catalyst (yellow arrows) (c). The MS signals on different channels collected during the H_2_‐TPR experiment for the spent catalysts (d and e). pXRD patterns of the *θ*‐Al_2_O_3‐900_ support, pristine catalyst, and spent samples after different reaction conditions (f).

Thermogravimetric analysis (TGA) is performed on the spent catalysts under different reaction conditions to quantify the extent of carbon deposition (Figures 4a and S11). The sample operated at DRM850 exhibits negligible weight loss relative to the fresh catalyst, whereas the catalyst tested at DRM800 displays a pronounced weight loss of approximately 15%, indicative of substantial coke formation. Differential scanning calorimetry (DSC) further reveals an exothermic peak above 600°C for the DRM800 spent catalyst, characteristic of the combustion of filamentous or nanotube‐like carbon species [39, 40, 41]. This observation is in good agreement with the high‐resolution STEM images (Figures 4b,c, and S12–S14), which reveal that the DRM850 spent catalyst contains either no detectable carbon deposits on most Ni nanoparticles or only a few layers of graphitic carbon. In contrast, the DRM800 spent sample clearly exhibits substantially large filamentous coke. Lattice fringe analysis further reveals metallic Ni with a fringe spacing of 0.18 nm, corresponding to the Ni (200) plane within the crystalline region highlighted by the pink dashed line. Notably, a less crystalline region is observed at the interface between the nanoparticle and the support, which likely corresponds to an oxidized Ni phase strongly interacting with the alumina support, as supported by the in situ XAS results. In addition, the unexpected lower agglomeration observed at 850°C compared with 800°C (Figures S12 and S13) can be attributed to the stabilizing effect of interstitial carbon in Ni nanoparticles, which alters the surface electronic structure and prevents sintering (suppressing Ostwald ripening) under reaction conditions [3, 42, 43]. Collectively, these findings indicate that elevated reaction temperatures effectively suppress carbon accumulation, whereas lower temperatures promote the formation of nanotube‐like carbonaceous species, ultimately leading to catalyst deactivation (Figure 4c).

Next, hydrogen temperature‐programmed reduction (H_2_‐TPR) coupled with mass spectrometry (MS) is employed to probe the reducible surface species of the spent catalyst. The corresponding MS traces (Figure 4d, *m*/*z* = 18) display multiple H_2_O evolution peaks centered at approximately 340°C and 840°C for DRM850 spent sample, and around 700°C for DRM800 spent sample. These reduction features lie between those of the NiO reference (268°C, Figure S15a) and the NiAl_2_O_4_ reference (863°C, Figure S15a), indicating the presence of Ni^2+^ species with different extents of interaction with the alumina support (vide infra) [44, 45, 46]. The lower‐temperature peak can be attributed to the reduction of more weakly bound or segregated NiO species, whereas the higher‐temperature peak reflects NiO species strongly interacting with the *θ*‐Al_2_O_3_ matrix, possibly forming a surface spinel‐like phase. Such strong metal–support interactions can significantly alter the reducibility and dispersion of Ni, thereby influencing the catalyst performance during DRM [44]. The presence of both low‐ and high‐temperature reduction peaks highlights the heterogeneity of the Ni environment in the spent catalyst and underscores the role of the support in stabilizing partially oxidized Ni species under reaction conditions [45, 47]. This TPR evidence for the presence of NiO species in the DRM850 spent sample is consistent with the HRTEM observations discussed above. Notably, multiple distinct CH_4_ peaks between 320°C to 600°C are detected during H_2_‐TPR of the DRM850 spent sample (Figure 4d, *m*/*z* = 16 and 15), indicating that the carbon present on this sample is sufficiently labile to react with H_2_ to form CH_4_ [48]. On the contrary, the DRM800 spent sample produced negligible CH_4_, despite exhibiting a substantially higher coke content (Figure 4e, *m*/*z* = 16 and 15). These differences strongly suggest that, at 850°C, carbon is incorporated into Ni, possibly forming small adsorbed islands and/or a carbide‐like phase, thereby generating more reactive carbon species, that can be hydrogenolysis to CH_4_ [42]. Conversely, at 800°C carbon is predominantly deposited on the external surface of Ni particles rather than inserted into the bulk, producing more inert carbonaceous deposits that accumulate on active sites, leading to the catalyst deactivation.

Synchrotron‐pXRD is also conducted on both fresh and spent catalysts to investigate phase evolution after DRM (Figure S4b‐d) [49]. The pXRD pattern of the DRM850‐spent sample does not exhibit distinct signals corresponding to NiO or NiAl_2_O_4_, suggesting that their presence is negligible. However, a noticeable shift in the Ni (200) reflection peak is observed (Figure 4f): this shift from 19.72° to 19.67° corresponds to a 0.25% expansion for the Ni (200) interplanar spacing, which can be attributed to carbon dissolution into the Ni lattice during DRM, forming a Ni─C solid solution within the face‐centered cubic (fcc) structure [3, 42, 43, 50]. These observations indicate that higher reaction temperatures (850°C) promote carbon incorporation into the Ni lattice and the formation of thin, graphitic carbon layers, both of which seem beneficial to catalyst stability. In contrast, lower temperatures (800°C) favor surface carbon accumulation and filamentous carbon growth, which are more likely to lead to catalyst deactivation.

**FIGURE 5:**
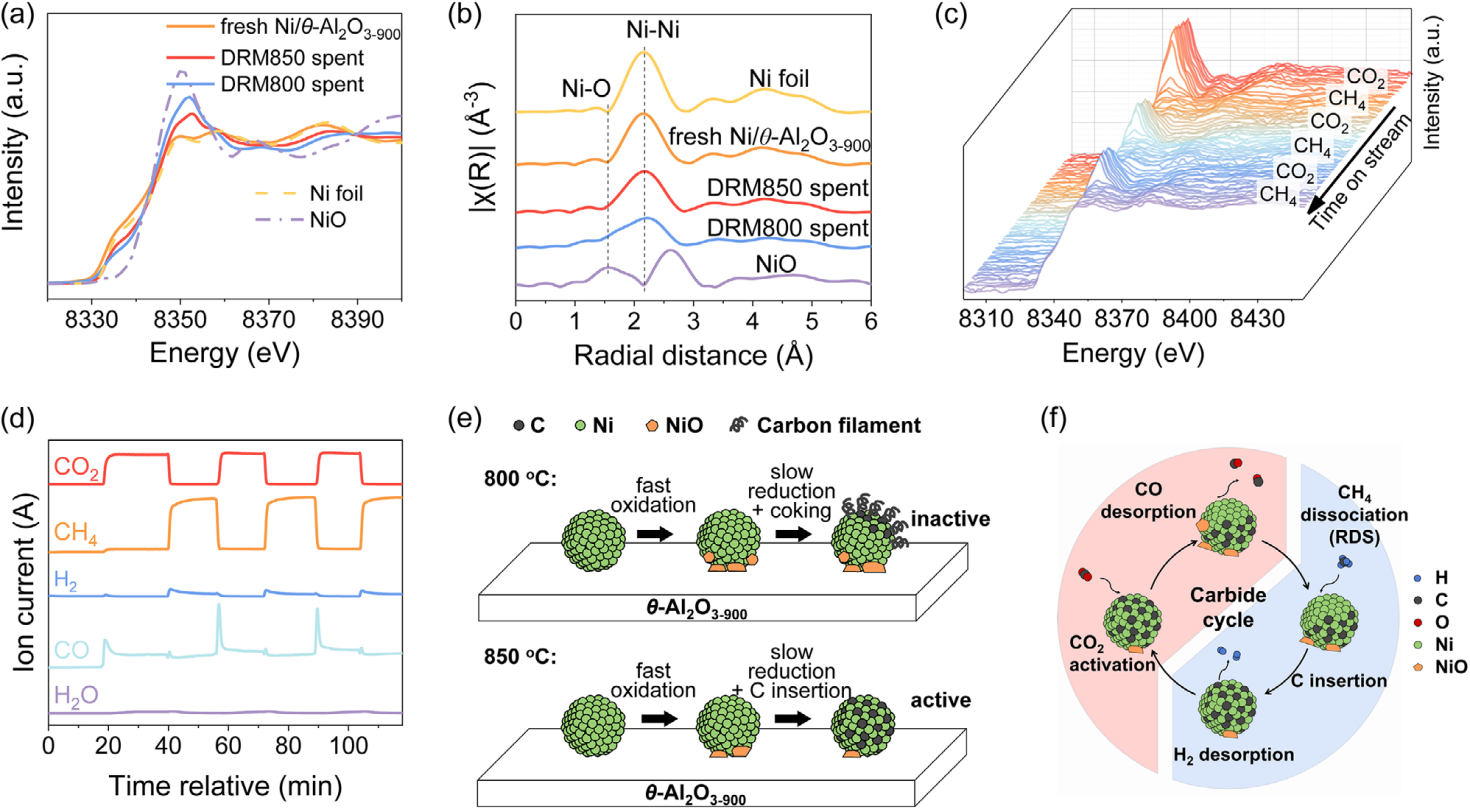
XANES spectra (a) and corresponding k^2^‐weighted Fourier transforms of EXAFS spectra at Ni K‐edge (b) under different conditions (pristine, DRM850 spent, and DRM800 spent). In situ Ni K‐edge XANES spectra (c) collected during gas switching, alternating CO_2_ and CH_4_ gas pulses at 850°C: significant Ni oxidation occurs upon introduction of CO_2_, followed by reduction under CH_4_. MS profiles of reactants and products signals during gas switching (d): large CO peaks correspond to CO_2_ reacting with surface‐bound carbon; continuous H_2_ production reflects CH_4_ pyrolysis. Schematic illustration of the induction period (e) and proposed carbide cycle mechanism (f).

We next also investigate the change of catalyst structure after different conditions (pristine, DRM850 spent, and DRM800 spent) via ex situ XAS experiments. After DRM at 850°C for 15 h, the Ni K‐edge XANES spectrum exhibits a slight shift of the absorption edge toward higher energy accompanied by an increase in white‐line intensity (Figure 5a). These spectral changes point to partial oxidation of the Ni phase under reaction conditions. Consistent with this observation, quantitative EXAFS fitting reveals a modest decrease in the Ni–Ni coordination number from 10.5 to 9.5, accompanied by an increase in the Ni–O coordination number from 0.3 to 2.1 (Figures 5b and S7 and Table S2). The LCF further indicates a reduction in the metallic Ni fraction from 86% to 77% (Figure S5b,d). Notably, satisfactory fitting requires inclusion of a Ni─C scattering path with a coordination number of 0.4 (Table S2), consistent with the carbon incorporation discussed above. These results suggest that, during the 15 h reaction following the induction period, a portion of the metallic Ni atoms becomes coordinated with oxygen species, while simultaneously incorporating carbon into the Ni lattice. In sharp contrast, the Ni K‐edge XANES spectra of the catalyst after DRM at 800°C for 15 h display a pronounced shift of the absorption edge to higher energy together with a marked increase in white‐line intensity, indicative of extensive oxidation of the Ni phase under relatively low temperature. EXAFS fitting analysis reveals a pronounced decrease in the Ni–Ni coordination number to 8.6, accompanied by an increase in the Ni–O coordination number to 3.5 (Figures 5b and S8, and Table S2). This evolution is consistent with the partial oxidation of Ni (approximately 40% of Ni oxidized, as shown in Figure S5c,e), with the additional Ni–O contribution arising from oxide species formed at the metal–support interface [51]. Taken together, these results demonstrate that at 800°C the metallic Ni is largely disrupted and transformed into oxidized Ni species, which would be expected to diminish catalytic performance. By contrast, under the higher‐temperature DRM conditions at 850°C, XANES and EXAFS analyses show only a minor shift of the edge position, a small increase in white‐line intensity, and relatively high Ni–Ni coordination numbers with additional Ni–C contributions and minimal Ni–O features. These findings indicate that the metallic Ni phase is largely preserved at 850°C, with only partial surface oxidation and carbon incorporation into the Ni lattice, paralleling the superior and sustained catalytic performance observed at elevated temperature.

### 2.5 Gas‐switching XANES‐MS Study

A gas‐switching experiment (Figure S16), monitored by XANES and MS, is carried out next to probe the dynamics evolution and reactivity of the catalyst (Figure 5c,d). After the pretreatment at 870°C and induction period at 850°C, the catalyst is subsequently flushed with Ar at 850°C, followed by alternating introduction of CO_2_ and CH_4_ for three cycles. The Ni K‐edge XANES spectra reveal that exposure to CO_2_ led to significant Ni oxidation, evidenced by an increase in the white‐line intensity. Upon switching to CH_4_, Ni rapidly reduces, restoring the metallic state [52, 53]. Notably, in the subsequent gas‐switching cycles, the white‐line intensity increase is substantially diminished, suggesting that the initial CH_4_ exposure induced a structure reconstruction that rendered Ni less susceptible to oxidation by CO_2_. The MS profiles (Figure 5d) display continuous H_2_ evolution during CH_4_ exposure, unambiguously confirming CH_4_ pyrolysis on the Ni surface with concomitant formation of H_2_ and carbonaceous surface species. As discussed above, this carbon readily migrates into the Ni lattice under reaction conditions to generate a carbide‐like phase, which is proposed to act as the catalytically active species during DRM. Notably, no CO signal is observed under CH_4_ flow, indicating that CO formation does not occur during the pyrolysis step. Upon switching the feed to CO_2_, pronounced CO peaks emerge, signifying that CO_2_ reacts with the carbon species stored on or within Ni to produce CO. The transient fluctuation in the H_2_O signal upon switching from CO_2_ to CH_4_ indicates the rapid reduction of partially oxidized Ni species at the initial stage of CH_4_ exposure; however, the limited amount and short duration of H_2_O formation suggest that this process is not dominant, with H_2_ being the primary product observed under CH_4_ flow. Furthermore, in the MS spectra obtained during gas switching experiments, the CO signal exhibits a pronounced increase immediately after the introduction of CO_2_, whereas the H_2_ signal shows a comparatively smaller increase followed by a stable plateau after CH_4_ is introduced. These observations suggest that the removal of interstitial carbon by CO_2_ proceeds more rapidly than the dissociation of CH_4_. Consequently, CH_4_ dissociation is likely the rate determining step in this catalytic cycle. As a result, we propose a dynamic carbide cycle mechanism during the DRM induction period (Figure 5e). Ni is rapidly oxidized by CO_2_ and slowly reduced by CH_4_, with carbon inserted into the Ni lattice at higher temperature, forming a carbide‐like phase—NiC_x_ (Figure 5f). This active phase acts as a dynamic carbon reservoir, facilitating a catalytic cycle in which CO_2_ activation removes C to form CO, while CH_4_ dissociation produces H_2_ and lattice C, regenerating the active phase. In contrast, at 800°C, carbon insertion process is less favorable, leading to surface carbon accumulation and catalyst deactivation (Figure 5e).

## 3 Conclusion

In summary, we have developed a high‐performance monometallic Ni catalyst supported on *θ*‐Al_2_O_3_ for DRM through a SOMC approach. The resulting Ni nanoparticles are highly dispersed and maintain a small average size of 5.3 ± 1.3 nm, even after high‐temperature reduction under 900°C. This catalyst exhibits a pronounced temperature‐dependent behavior under DRM conditions, with significant structural and mechanistic differences observed within a narrow 50°C range. Detailed mechanistic investigations, through in situ XAS and ex situ synchrotron XRD analyses, reveal dynamic induction process involving an initial rapid oxidation of Ni upon CO_2_ exposure, followed by CH_4_ pyrolysis on the Ni surface. This reduction step not only regenerates metallic Ni but also incorporates carbon atoms into the Ni lattice at 850°C, forming a carbide‐like NiC_x_ phase. At lower temperature (800°C), this carbon insertion process is significantly less effective, leading to surface carbon accumulation and catalyst deactivation. Gas‐switching experiments support the operation of a carbide cycle at 850°C, in which CH_4_‐derived carbon is incorporated into the Ni lattice and subsequently removed by CO_2_ to generate CO. This mechanism minimizes carbon buildup on the surface and contributes to the excellent coking resistance and long‐term stability of Ni/*θ*‐Al_2_O_3‐900_ under DRM conditions. This work highlights the importance of induction dynamics and structural evolution in DRM catalysis and provides mechanistic insight that could inform the design of the next‐generation Ni‐based catalysts for industrial methane reforming processes.

## Conflicts of Interest

The authors declare no conflicts of interest.

## Supporting information




**Supporting File 1**: anie71925‐sup‐0001‐SuppMat.pdf.

## Data Availability

The data that support the findings of this study are available from the corresponding author upon reasonable request.
