# Supplementary material for: Initiation: A Critical Step for High Activity and Stability in Ni‐Based Methane Dry Reforming Catalysts Supported on θ‐Al2O3
Source: Angew Chem Int Ed Engl. 2026 Mar 25;65(19):e1716058. doi: 10.1002/anie.1716058 (PMC13134598; doi:10.1002/anie.1716058)
Supplement: Supplementary file 1 — Supporting File 1: anie71925‐sup‐0001‐SuppMat.pdf. [file ANIE-65-e1716058-s001.pdf]

Supporting Information

Initiation: a critical step for high activity and stability in Ni-based methane dry reforming catalysts supported on θ-Al_2O_3

Wei Wang,[a] Milivoj Plodinec,[a,b] Wei Zhou,*[a] and Christophe Copéret*[a]

[a]*Department of Chemistry and Applied Biosciences, ETH Zürich, CH-8093 Zurich, Switzerland*
[b]*ScopeM, ETH Zürich, CH-8093 Zurich, Switzerland*
*Corresponding author: weizhou@ethz.ch, ccoperet@ethz.ch

Experimental and details

General procedure

All operations of precursor synthesis and catalysts preparation were performed under an argon atmosphere in an M. Braun glove box or using standard Schlenk techniques. After preparation, all materials were stored in an argon filled glovebox. Deuterated Benzene (C_6D_6) was obtained via vacuum distillation from purple Na^0/benzophenone. Pentane, Benzene, and Toluene were dried using an M. Braun SPS 800 solvent purification system where columns used for purification were packed with activated copper and alumina. All solvents were further degassed via three freeze-pump-thaw cycles and stored over 4 Å molecular sieves after being transferred to a glove box.

Preparation of θ-$Al_2O_{3\text{-}x}$: θ-Al_2O_3 was synthesized via the staged calcination method as reported.[1] Typically, η-Al_2O_3 was prepared according to a literature procedure[2]. θ-Al_2O_3 was prepared by calcination of η-Al_2O_3 at 950 °C (2 °C·min^{-1}) in air for 12 h. Then the freshly made θ-Al_2O_3 was dehydroxylated at different temperatures for 12 h (5 °C·min^{-1}) under high vacuum (10^{-5} mbar) to produce θ-$Al_2O_{3\text{-}X}$ (X denotes the temperature for thermal treatment). The surface area of the oxide support was determined from nitrogen physisorption isotherms (-196 °C) and application of the BET method. The surface hydroxyl (Al−OH) density is quantified by titration with $Mg(CH_2Ph)_2(THF)_2$, following eq 1.

$$\text{Al–OH} + \text{MgBn}_2 = \text{Al–O–MgBn} + \text{Toluene} \qquad \text{(eq 1)}$$

Synthesis of the molecular precursor: Tetramethylethylenediamine(dimethyl)nickel(II) [(tmeda)$NiMe_2$] was prepared according to adapted literature protocols.[3] (tmeda)$NiMe_2$ was synthesized from (tmeda)$Ni(acac)_2$ according to adapted literature protocols.[4] Typically, to a solution of (tmeda)$Ni(acac)_2$ (1865.6 mg, 5 mmol) in diethyl ether (90 mL) in a 500 mL Schlenk tube was added methyllithium (6.25 mL, 1.6 M in diethyl ether, 10 mmol) at -78 °C within 5 min with stirring. The temperature was raised to -30 °C by addition of solvent to the cooling bath, and stirring was continued for 30 min at -30 °C, while the initially blue mixture gradually turned yellow-brown. The cooling bath was removed, and the mixture was evaporated to dryness while it was gradually warmed to room temperature. Cyclohexane (200 mL) was added, and the mixture was sonicated in an ultrasound bath and then cannula transferred into a Schlenk frit. After filtration, the residue was washed with additional portions of cyclohexane (3 × 15 mL), and the yellow filtrate was evaporated to dryness to yield (tmeda)$NiMe_2$ as a pale yellow to yellow-brown solid (579 mg, 56.5%) which contains ca. 1−2 mol % of lithium acetylacetonate as a trace impurity. Solution ^{1}H NMR (400 MHz, C_6D_6, 298 K): δ 1.961 (s, 12 H, NCH_3), 1.386 (s, 4 H, NCH_2), −0.470 (s, 6 H, $NiCH_3$). ^{13}C NMR (100 MHz, C_6D_6, 298 K): δ 58.4 (NCH_2), 47.0 (NCH_3), −12.6 ($NiCH_3$).

Preparation of Ni/θ-$Al_2O_{3\text{-}900}$: A nominal loading of 1.1 wt% of Ni was applied. Typically, a yellow solution of (tmeda)$NiMe_2$ (15.6 mg, 0.076 mmol) in 3 mL of benzene was added dropwise to a stirred slurry of θ-$Al_2O_{3\text{-}900}$ (400 mg) in 10 mL benzene leading to an instant discoloration of the supernatant,

and a color change of θ-$Al_2O_{3\text{-}900}$ from white to grey after 4 h. After deposition, the supernatant was removed, and the deposited support was washed three times with benzene (3 x 5 mL). The supernatant plus the benzene washings were analyzed via ^1H NMR revealing 91% grafting efficiency according to remaining precursor in solution. After drying all volatiles under vacuum, the samples were reduced under pure hydrogen flow at 900 °C (1 °C·min^{-1}) for 8 h. After outgassing under a high vacuum (10^{-5} mbar), the catalysts were stored in a glovebox (Argon).

Catalytic dry reforming of methane. The catalytic performance tests were conducted in a fixed-bed (PID Eng&Tech) quartz reactor with an inner diameter of 9 mm. Typically, 20mg of catalyst was mixed with 200 mg of SiC and packed in the reactor in glovebox. The tests were performed at temperatures between 700 °C to 850 °C. After the pretreatment under 870 °C in H_2 atmosphere for 1 h, the total flow rate of the feed gas was introduced in 20 or 50 sccm (GHSV of 60 000 or 150 000 mL·g_{cat}^{-1}·h^{-1}; composition: 40% CH_4, 40% CO_2, and 20% N_2). The effluent gases were analyzed via online gas chromatography (Thermofisher 1300 equipped with Hayesep N, 60-80, 0.25 m x 1/16" SS + Hayesep Q, 60-80, 1 m x 1/16" SS) two thermal conductivity detectors (TCD) for CH_4, CO_2, CO, N_2 and H_2. GC data was collected in increments of 16 min. The CH_4 conversion and H_2/CO ratio are calculated using the following set of equations:

$$Conversion_{CH_4} = \frac{F_{CH_4}^{\text{in}} - F_{CH_4}^{\text{out}}}{F_i^{\text{in}}} \times 100\%$$

$$Conversion_{CO_2} = \frac{F_{CO_2}^{\text{in}} - F_{CO_2}^{\text{out}}}{F_i^{\text{in}}} \times 100\%$$

$$\frac{H_2}{CO} = \frac{F_{H_2}^{\text{out}}}{F_{CO}^{\text{out}}}$$

Catalyst characterization:

Fourier-Transform Infrared (FTIR): FTIR spectroscopy experiments were performed on self-supporting wafers using a Bruker Alpha FT-IR spectrometer in transmission mode (32 scans, 2 cm^{-1} resolution) under air-free conditions. Spectra are normalized to the Al-O-Al overtone peak maximum at 1522 cm^{-1} for all the materials.

Nuclear magnetic resonance (NMR): Solution NMR spectra were recorded using a Bruker 200 MHz spectrometer. Solid state NMR spectra were taken either in a 700 MHz (3.2mm, HX probe) or in a 400 MHz (4.0mm, HX probe) Bruker spectrometers and referenced to adamantane by setting the ^1H signal to 1.8 ppm.

Elemental Analysis (EA): Elemental Analysis was performed by Mikroanalytisches Labor Pascher (an der Pulvermühle 1, D-53424 Remagen, Germany) using ICP-AES.

Transmission electron microscopy (TEM): Catalysts morphologies were assessed by (scanning) transmission electron microscopy (S)TEM using a double aberration-corrected JEOL GRAND ARM "Vortex" operated at 300 kV and a JEOL JEM F200 operated at 200k V, both microscopes are

available within the ScopeM facility at ETH Zürich. All the samples were prepared under air-free conditions inside the glovebox by dry drop-casting onto an ultrathin carbon support film on a lacey carbon 400-mesh copper grid. The samples were transferred to the microscope using a Gatanvacuum transfer holder. The particle size distributions were determined by counting above 100 individual particles in each sample, while the average size and standard deviation are determined by fitting the data with a normal distribution function.

Thermogravimetric Analysis / Temperature Programmed Oxidation: Thermogravimetric analyses (TGA) and differential scanning calorimetry (DSC) coupled with mass spectrometry (MS) of the fresh and spent catalysts were performed using Netzsch TGA/DSC/MS instrument. The air-exposed samples were flushed with Ar (50 mL·min^{-1}) at 150 °C as a pretreatment. Subsequently, the analysis was carried out under a flow of 5% O_2/Ar (50 mL·min^{-1}) heating 20-30 mg of samples from 50 °C to 800 °C at 5 °C/min. Note that additional background measurements were performed with an empty crucible. The evolved gas was analyzed by MS to track the formation of CO_2 (m/z=44). The obtained MS signals for CO_2 were normalized to sample weight and Ar (m/z=44), which show the stable intensity over the measurements.

Powder X-Ray Diffraction (pXRD): pXRD pattern for the support was acquired on a Bruker AXS D8 Advance diffractometer operated at 50 kV and 40 mA at 2θ (Cu K_α) = 10-80°. The scanning step size and speed were set to 0.495° and 10 s per step, respectively.

Synchrotron-XRD: Synchrotron-XRD patterns for the spent samples were collected at BM01A (λ = 0.60325 Å) of The Swiss-Norwegian Beamlines (SNBL) located at the European Synchrotron Radiation Facility (ESRF) in Grenoble, France.[5] The detector distance was set to either 200 or 500 mm, with a collection time of 300 s to ensure high-quality diffraction patterns.

***In situ* X-ray absorption spectroscopy (XAS) experiments:** *In situ* XAS experiments were measured at BM31 of the SNBL. All samples and sample containing cells were exclusively handled and prepared in Argon-filled glove boxes. All gases used (Ar, H_2, CO_2, and CH_4) are purified using gas traps filled with 3Å molecular sieve and Cu-based catalyst pellets. *In situ* cells are built from a stainless-steel U-shaped frame, in which a quartz glass capillary (1.0 mm outer diameter, 0.01 mm wall thickness) is glued in place, using epoxy-glue. Swagelok 1/8-inch.three-way are used to allow for air free transport and mounting. A catalyst bed of ca. 1 cm length was used. The powdered sample was secured using quartz wool plugs at each end and loaded into a cell. Ni K-edge spectra were collected in transmission mode using a double crystal Si (111) monochromator. The ionization chambers were filled with a gas mixture optimized for the edge energy and the required path length. A secondary reference for energy calibration was used (Ni-foil). For acquisition of EXAFS spectra, beam energies ranging from 8.23-9.33 keV for the Ni K-edge was collected. 5 scans were averaged for Ni K-edge to obtain a sufficient quality suitable for structural analysis. The acquisition time for XANES and EXAFS spectra at the Ni K-edge was ca. 1 minutes. Flow rates (Ar, H_2, CO_2, and CH_4) were controlled using mass-flow controllers (Bronkhorst), and the pressure was 1 bar. Throughout

the experiments, a total flow of 10 mL min^{-1} was maintained, and the outlet gas was monitored using a mass spectrometer. The temperature for H_2 reduction (870 °C) and DRM (800 to 850 °C) were controlled using a SiC heating element. In a typical experiment, EXAFS spectra were first collected after the sample was mounted. Subsequently, the air in the line was purged by Ar before a H_2 pretreatment was carried out under 10 mL min^{-1} of H_2 for 30 min at 870 °C (300 °C h^{-1} ramp), while continuously collecting XANES spectra. After cooling to room temperature (or < 50 °C) under a flow of H_2, EXAFS spectra of the reduced samples were collected. This was followed by heating to the reaction temperature (850 °C) under a flow of 10 mL·min^{-1} of a H_2 (300 °C h^{-1} ramp). Subsequently, the gas composition was switched to a CO_2/CH_4/Ar (2:2:1) mixture and the conditions were retained for 1 h to track the dynamic change of Ni XANES. Notably, the XANES spectra were continuously collected during the entire reaction protocol. During the gas switching experiment and temperature change experiment, the duration for each atmosphere and temperature was 20-30 min. After DRM test, the gas feed was switched to Ar and the sample was cooled to room temperature for a collection of post-DRM EXAFS spectra.

***Ex situ* XAS experiments:** Ni K edge XAS were also measured at Paul Scherrer Institute (PSI) at the Debye beamline at SLS2.0 operating in top-up mode at 400 mA ring current and 2.7 GeV. The beam originating from the 2.1 T bending magnet source was collimated with a Si mirror at 2.38 mrad and subsequently monochromatized by a LN cooled channel cut Si 311 qexafs monochromator. The beam was further focused with a Rh coated toroidal mirror yielding an Xray beam size on sample of 100 um x 100 um. Gas filled ionization chambers, 1.5 bar N_2, were used to measure the incident and transmitted beam intensity. The sample absorption was measured simultaneously with a Ni reference foil. Sample XAS data was acquired in fluorescence geometry using a PIPS detector. Demeter software (0.9.26) from the Ifeffit software package (Version 1.2.11) was used for the XAS data analysis.[6]

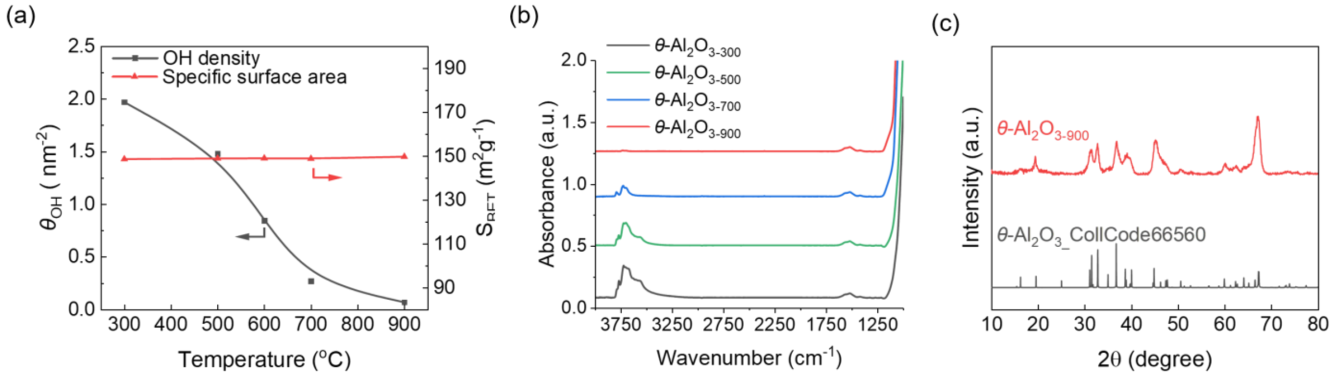

Figure S1. (a) The effect of thermal treatment on θ-Al_2O_3: left axis – hydroxy group coverage θ_{OH} measured by titration; right axis – specific surface area S_{BET} of θ-Al_2O_3 after thermal treatment at different temperatures. (b) The IR spectra of θ-Al_2O_3 after thermal treatment at different temperatures. (c) The pXRD pattern of θ-$Al_2O_{3\text{-}900}$ after thermal treatment at 900 ^{o}C under high vacuum.

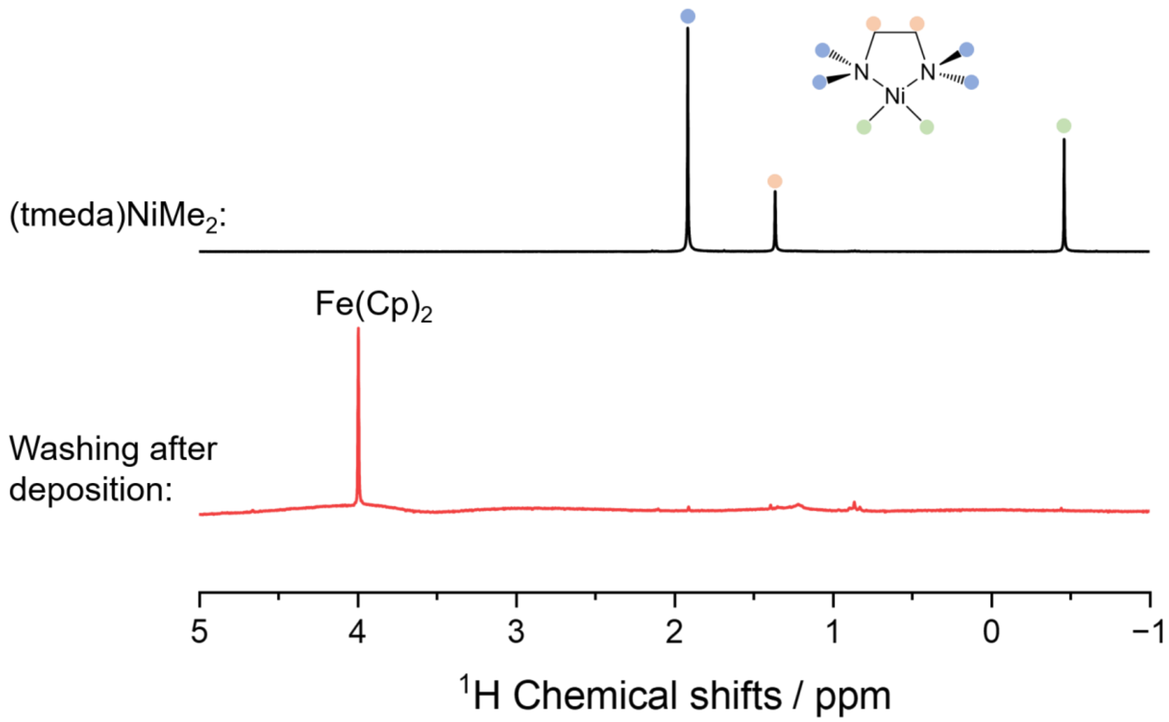

Figure S2. ^{1}H-NMR spectrum of (tmeda)NiMe$_2$ and the washing after deposition. The Fe(Cp)$_2$ is used as internal standard for quantification analysis for the residual precursor in the washing.

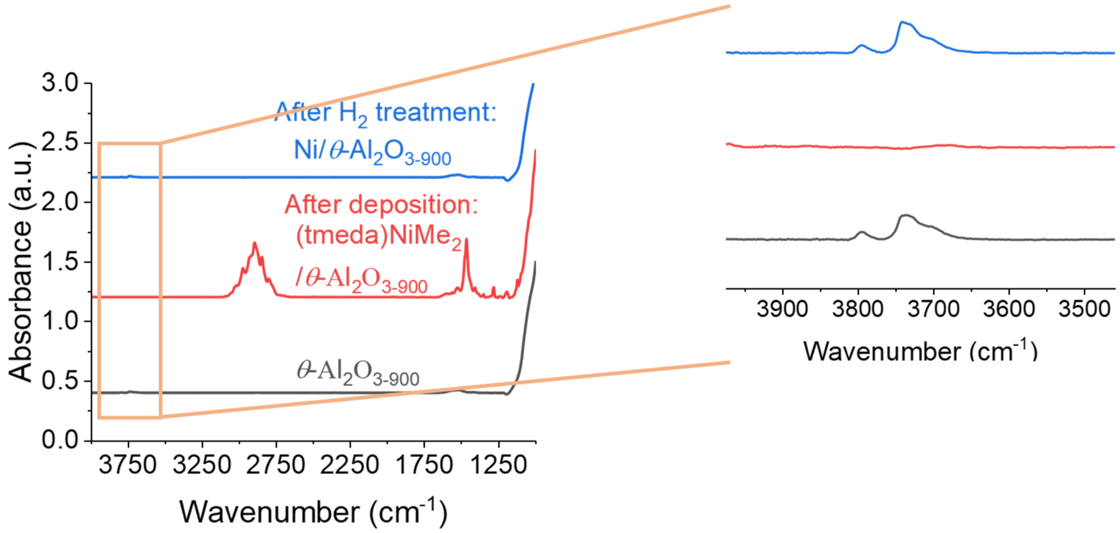

Figure S3. The FTIR spectra before deposition, after deposition, and after H_2 treatment during the synthesis process (right panel: zoom into small region).

Table S1. Consuming rates of CH_4 over Ni-based catalysts in DRM.

catalyst	Ni size (nm)	reaction conditions			CH_4 conversion (%)	CH_4 consumption rate ($mmol \cdot g_{Ni}^{-1} \cdot s^{-1}$)	reference
		T (°C)	GHSV ($L \cdot h^{-1} \cdot g_{cat}^{-1}$)	$CH_4/CO_2/N_2$			
Ni/θ-$Al_2O_{3\text{-}900}$ (after induction period)	5.3	850	150	2/2/1	81	60.2	This work
		800	150	2/2/1	67	51.0	
		750	150	2/2/1	52	39.6	
		700	150	2/2/1	35	26.7	
Ni/θ-Al_2O_3	20	800	60	1/1/3	96	2.4	[7]
Ni/γ-Al_2O_3	8.9-11.3	650	25	1/1/2	10	9.4	[8]
Ni/γ-Al_2O_3	6.2	800	108	1.5/1.5/7	95	3.8	[9]
Ni/$MgNiO_2$	4.7	700	60	1/1/0	71	1.1	[10]
NiSr/Al_2O_3	31.4	800	96	1/1/1	95	3.8	[11]
$NiCuFeAl_2O_4$	10.0	800	34	1/1/55	97	0.3	[12]
NiCo/Al_2O_3	6.0	800	300	9/9/2	76	84.7	[13]
$Ni_3ZnC_{0.7}/Al_2O_3$	-	650	18	1/1/0	69	0.1	[14]
NiV/MgAl	6.6	700	480	1/1/2	54	4.4	[15]
NiGaC/MgO	7	600	54	1/1/1	48	0.8	[16]

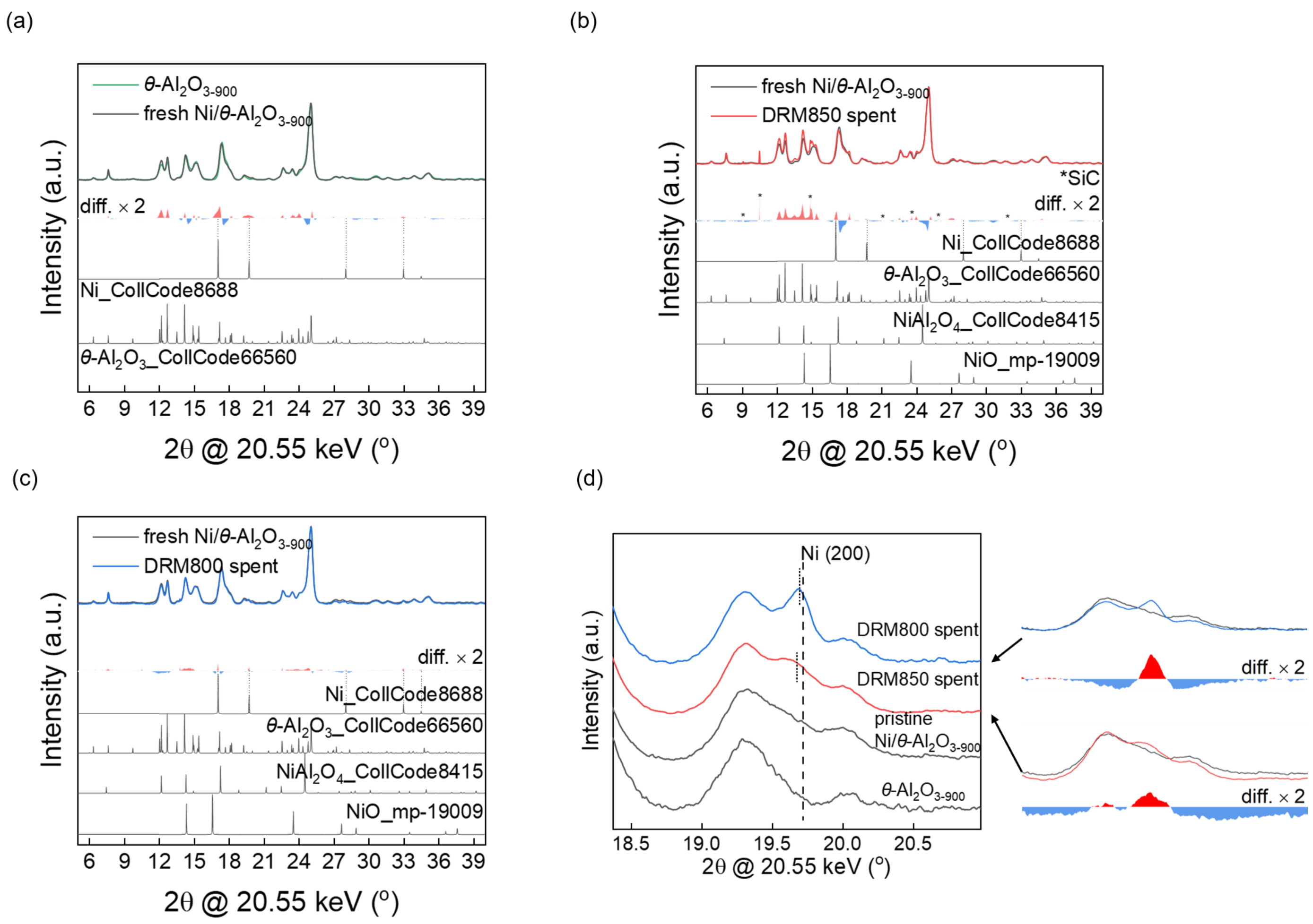

Figure S4. Comparations of the synchrotron-powder XRD patterns: the support with the fresh Ni/θ-Al_2O_3 (a); the DRM850 spent sample with the fresh Ni/θ-$Al_2O_{3\text{-}900}$ (b); the DRM800 spent sample with the fresh Ni/θ-$Al_2O_{3\text{-}900}$ (c); and the zoom in area around Ni (200) peak (left panel) (d). Peak positions are determined by pseudo-Voigt fitting of the subtraction spectra obtained by subtracting the fresh sample from the spent samples (right panel) (d).

(a)

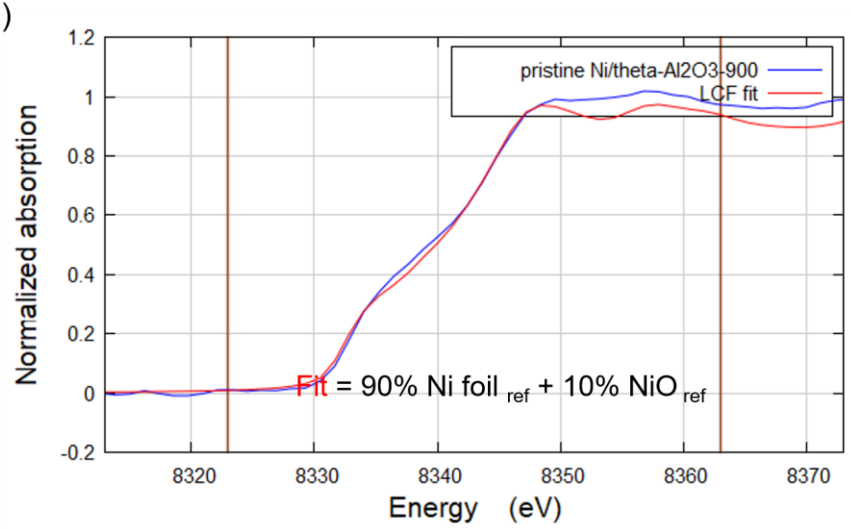

pristine Ni/theta-Al2O3-900
LCF fit
Fit = 90% Ni foil ref + 10% NiO ref
Normalized absorption
Energy (eV)
1.2
1
0.8
0.6
0.4
0.2
0
-0.2
8320
8330
8340
8350
8360
8370

(b)

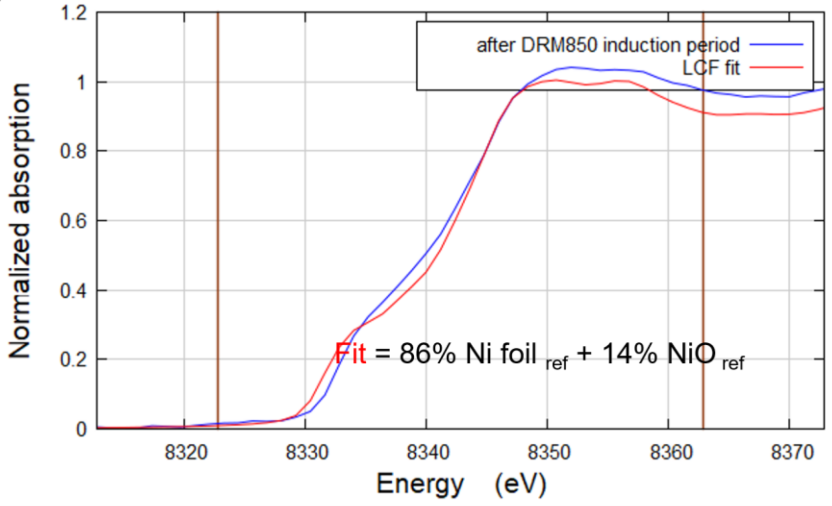

after DRM850 induction period
LCF fit
Fit = 86% Ni foil ref + 14% NiO ref
Normalized absorption
Energy (eV)
1.2
1
0.8
0.6
0.4
0.2
0
8320
8330
8340
8350
8360
8370

(c)

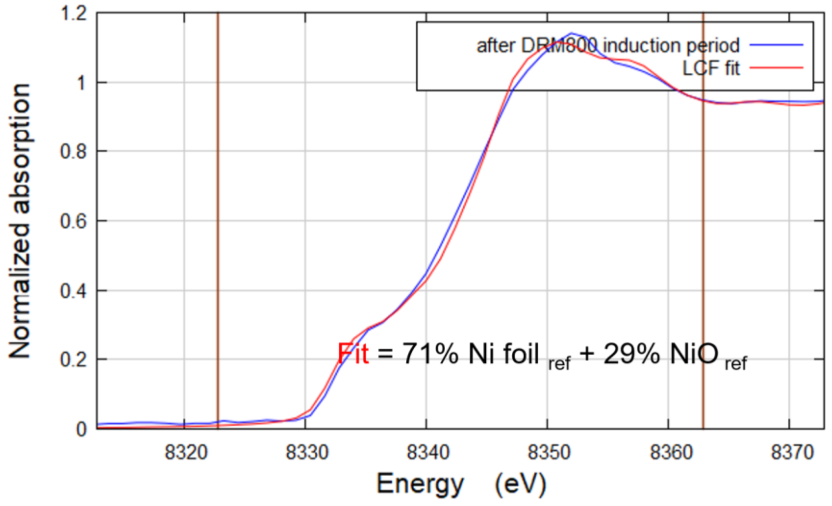

after DRM800 induction period
LCF fit
Fit = 71% Ni foil ref + 29% NiO ref
Normalized absorption
Energy (eV)
1.2
1
0.8
0.6
0.4
0.2
0
8320
8330
8340
8350
8360
8370

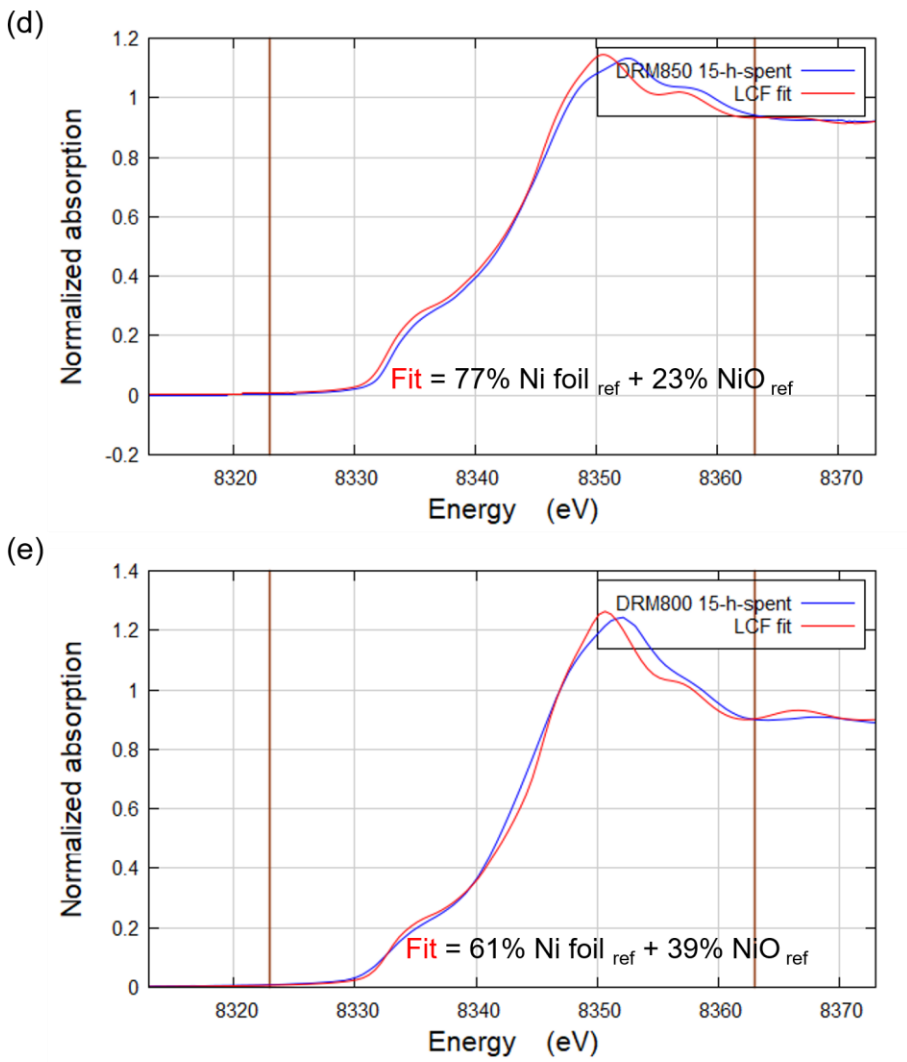

Figure S5. XANES linear combination fitting using Ni foil and NiO components for pristine Ni/*θ*-$Al_2O_{3\text{-}900}$ at 850 °C during the H_2 treatment (a); Ni/*θ*-$Al_2O_{3\text{-}900}$ after induction period at 850 °C (b); Ni/*θ*-$Al_2O_{3\text{-}900}$ after induction period at 800 °C (c); Ni/*θ*-$Al_2O_{3\text{-}900}$ after DRM850 for 15 h (d); Ni/*θ*-$Al_2O_{3\text{-}900}$ after DRM800 for 15 h (e).

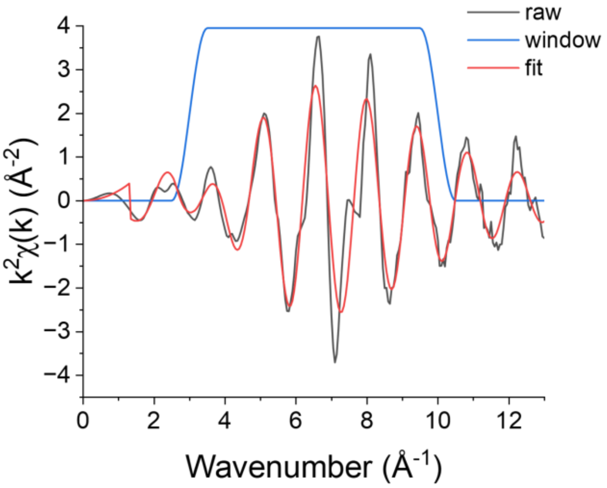

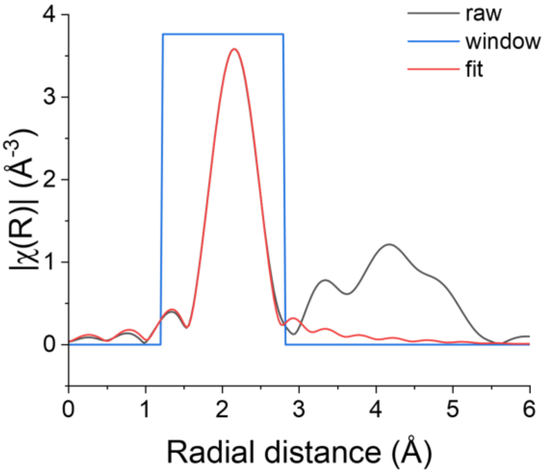

Figure S6. EXAFS fitting for pristine Ni/*θ*-$Al_2O_{3\text{-}900}$. (top) k-space with raw (black) and fitted (blue) data. Window (red) 3.0-10 Å^{-1}, k-weight = 2, Hanning window, dk = 1; (bottom) R-space with raw (black) and fitted (blue) data. Window (red) 1.2-2.8 Å, k-weight = 2, Hanning window, dr = 0. Fit summarized in Table S1.

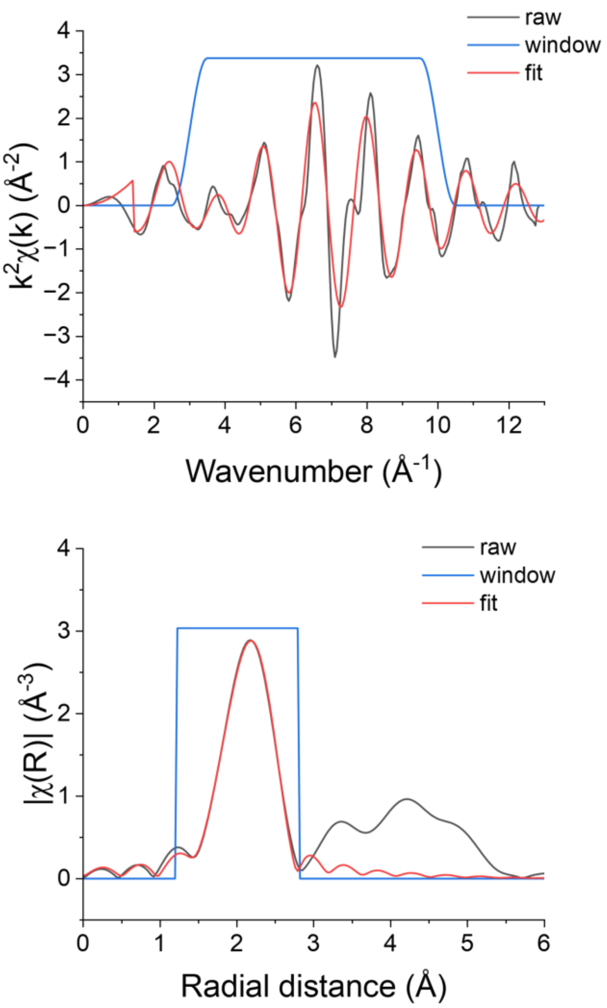

Figure S7. EXAFS fitting of the spent Ni/*θ*-$Al_2O_{3\text{-}900}$ after 15 h under DRM850. (top) k-space with raw (black) and fitted (blue) data. Window (red) 3.0-10 Å^{-1}, k-weight = 2, Hanning window, dk = 1; (bottom) R-space with raw (black) and fitted (blue) data. Window (red) 1.2-2.8 Å, k-weight = 2, Hanning window, dr = 0. Fit summarized in Table S1.

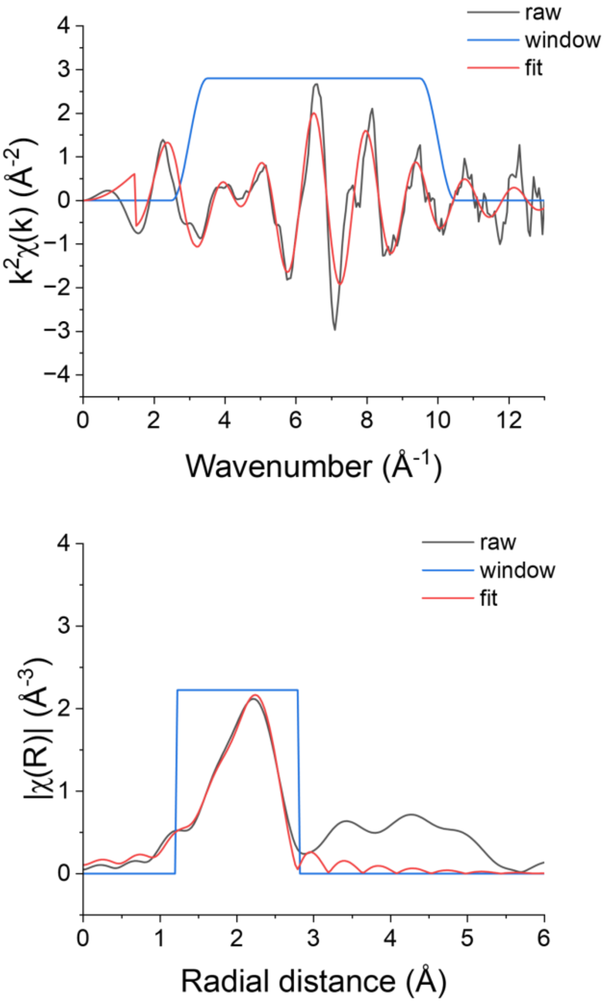

Figure S8. EXAFS fitting of the spent Ni/*θ*-$Al_2O_{3\text{-}900}$ after 15 h under DRM800. (top) k-space with raw (black) and fitted (blue) data. Window (red) 3.0-10 $Å^{-1}$, k-weight = 2, Hanning window, dk = 1; (bottom) R-space with raw (black) and fitted (blue) data. Window (red) 1.2-2.8 Å, k-weight = 2, Hanning window, dr = 0. Fit summarized in Table S1.

Table S2. Summary of Ni K-edge fitting results of Ni/θ-$Al_2O_{3\text{-}900}$ under different conditions. [a]

Conditions	Path	CN[b]	σ^2 (Å^2)[c]	ΔE (eV)[d]	R (Å)[e]
Pristine	Ni-Ni	10.5(0.8)	0.0061(0.0007)	6.6(0.8)	2.48(0.01)
	Ni-O	0.3(0.3)	0.0061(0.0007)	6.6(0.8)	1.92(0.08)
DRM850 spent	Ni-Ni	9.5(0.6)	0.0069(0.0007)	7.8(0.7)	2.49(0.01)
	Ni-O	2.1(0.4)	0.0069(0.0007)	7.8(0.7)	2.01(0.01)
	Ni-C	0.4(0.4)	0.0069(0.0007)	7.8(0.7)	1.73(0.07)
DRM800 spent	Ni-Ni	8.6(2.0)	0.0083(0.0021)	8.0(2.0)	2.50(0.01)
	Ni-O	3.5(0.9)	0.0083(0.0021)	8.0(2.0)	2.03(0.02)

[a] $3.0 < k < 10$; S_0^2 was fixed as 0.80504; $1.2 < R < 2.8$ for the pristine sample and DRM850 spent sample; k-weight = 2. [b] coordination number. [c] Debye-Waller parameter. [d] energy correction factor. [e] interatomic distance.

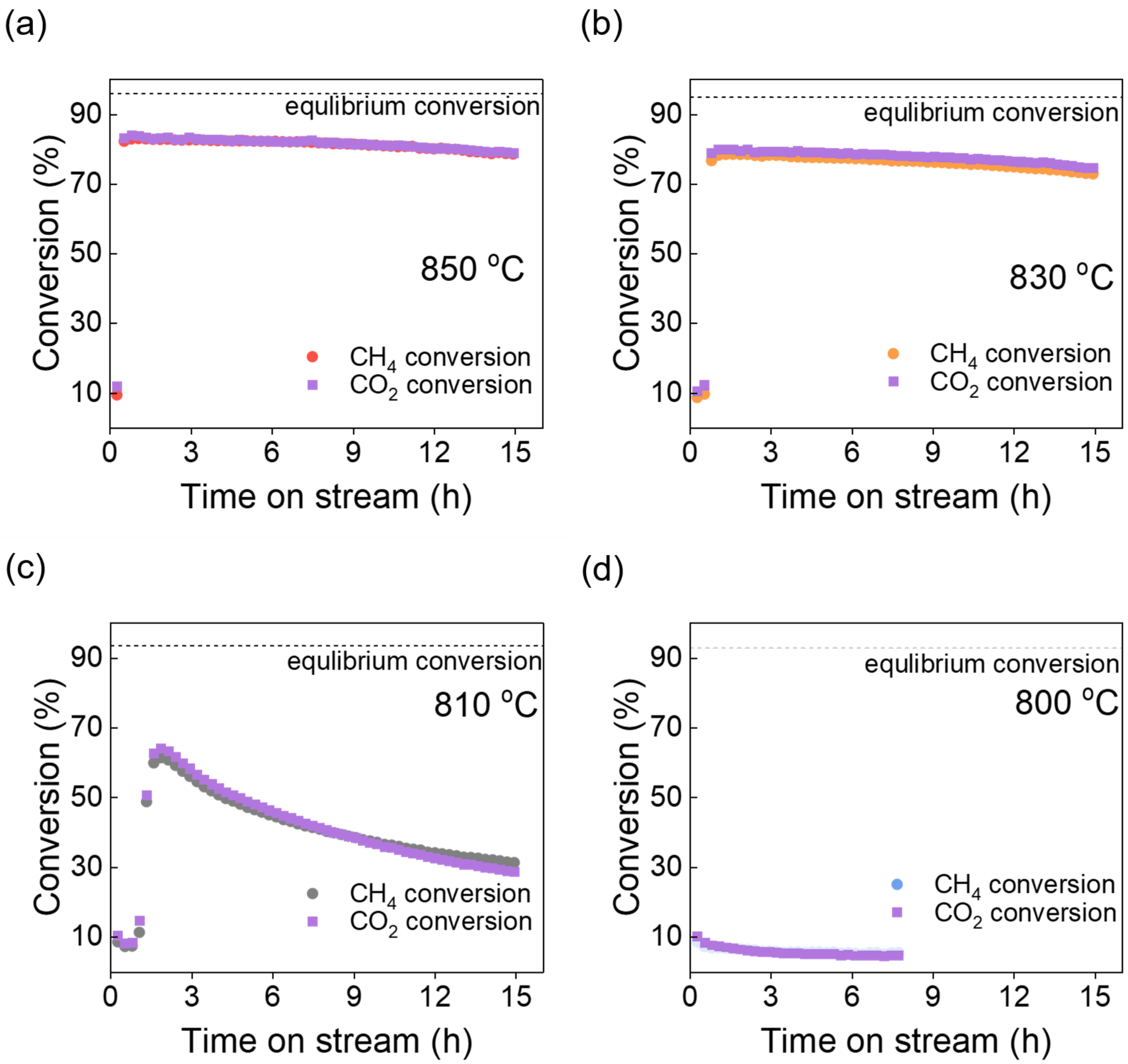

Figure S9. The CH_4 and CO_2 conversions over Ni//*θ*-$Al_2O_{3\text{-}900}$ during DRM under corresponding temperatures.

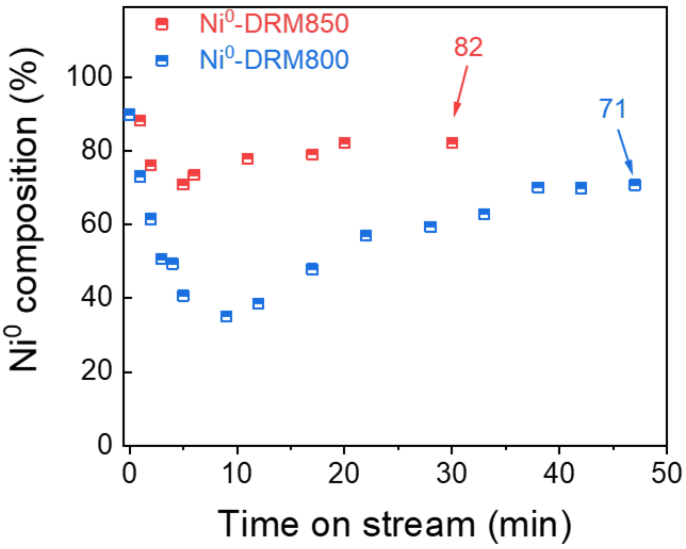

Figure S10. LCF analysis showing the dynamic evolution of Ni^0 species during the induction period under different DRM temperatures.

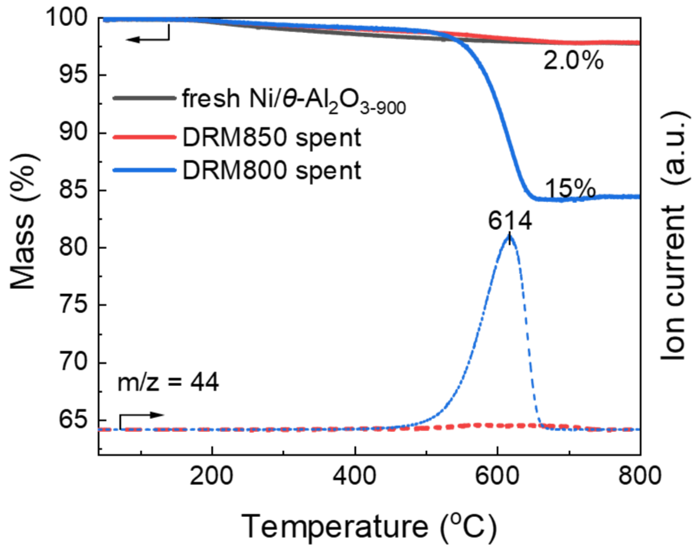

Figure S11. TGA profiles with concurrent CO_2 (m/z = 44) evolution monitored by MS for spent samples obtained under different conditions. (DRM850 spent, and DRM800 spent).

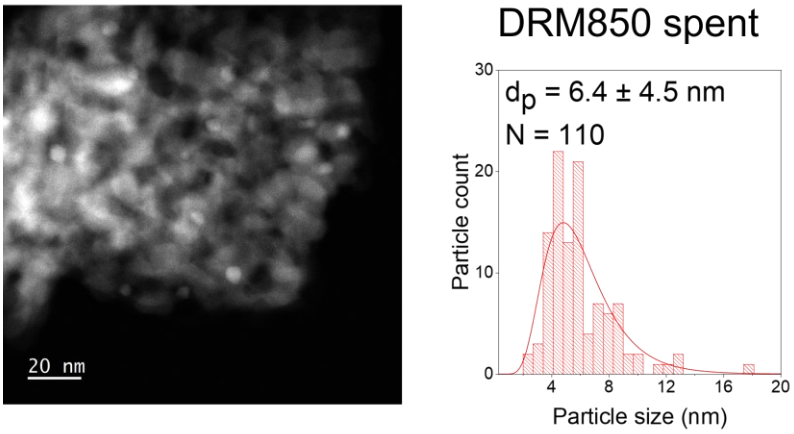

Figure S12. STEM analysis of DRM850 spent catalyst. [Left] High-angle annular dark-field STEM (HAADF-STEM) overview image; [Right] Particle size distribution.

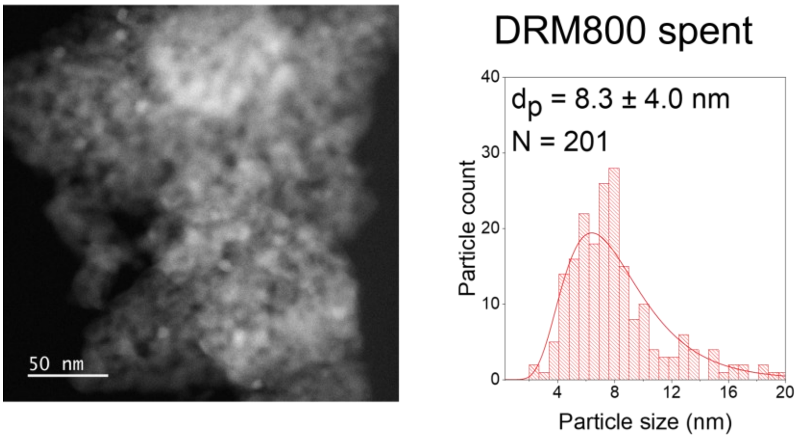

Figure S13. STEM analysis of DRM800 spent catalyst. [Left] HAADF-STEM overview image; [Right] Particle size distribution.

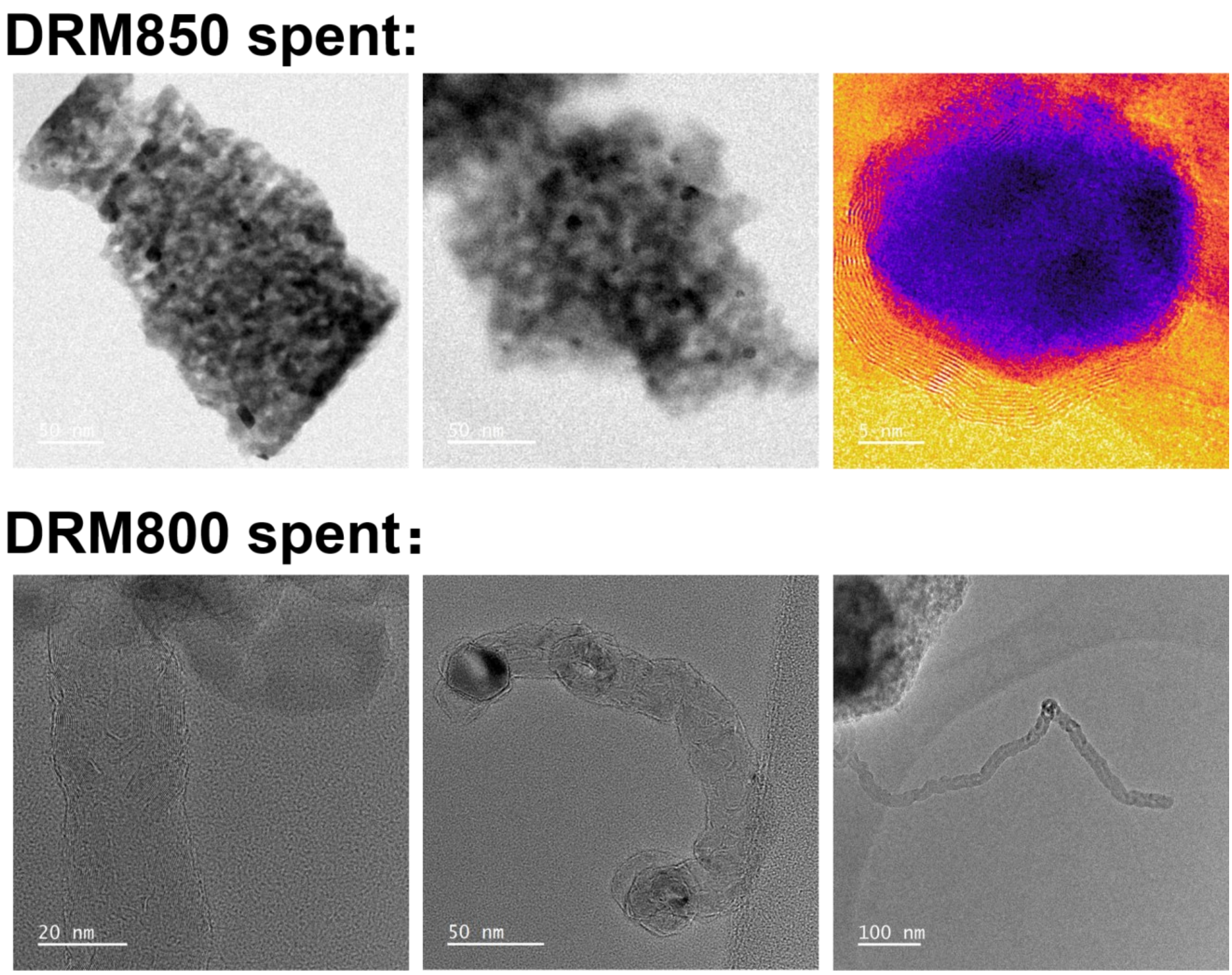

Figure S14. STEM analysis of spent catalyst. [Top left and middle] Annular bright-field (ABF) image and [Top right] False colorized ABF-STEM of DRM850 spent catalyst; [Bottom left and middle] High-resolution TEM and [Bottom right] TEM images of DRM800 spent catalyst.

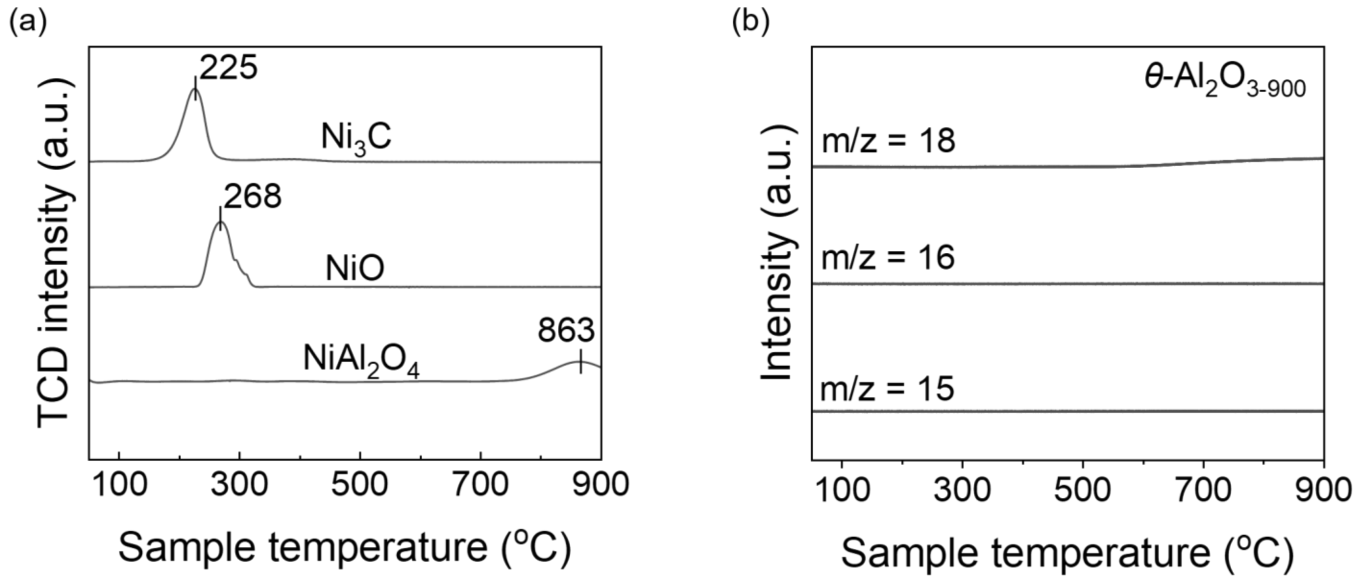

Figure S15. TCD signal during H_2-TPR experiments for the references (Ni_3C, NiO, and $NiAl_2O_4$) (a); and the MS spectra on different channels during H_2-TPR experiment for the pure support θ-$Al_2O_{3\text{-}900}$ (b).

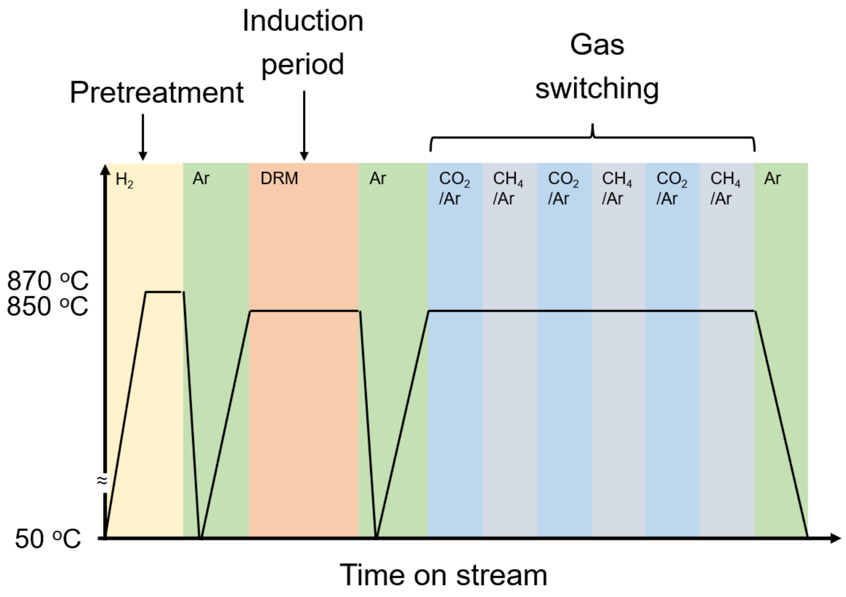

Figure S16. The procedure of *in situ* XAS analysis of gas switching experiment.

References:

[1] J. Meyet, A. Ashuiev, G. Noh, M. A. Newton, D. Klose, K. Searles, A. P. van Bavel, A. D. Horton, G. Jeschke, J. A. van Bokhoven, C. Coperet, *Angew. Chem. Int. Ed.* **2021**, *60*, 16200-16207.

[2] D. Maciver, *J Catal* **1963**, *2*, 485-497.

[3] A. Zeller, E. Herdtweck, T. Strassner, *Inorganic Chemistry Communications* **2004**, *7*, 296-301.

[4] D. Gioffrè, L. Rochlitz, P. A. Payard, A. Yakimov, C. Copéret, *Helv. Chim. Acta* **2022**, *105*, e202200073.

[5] V. Dyadkin, P. Pattison, V. Dmitriev, D. Chernyshov, *J. Synchrotron Radiat.* **2016**, *23*, 825-829.

[6] B. Ravel, M. Newville, *J Synchrotron Radiat* **2005**, *12*, 537-541.

[7] H.-S. Roh, K.-W. Jun, S.-C. Baek, S.-E. Park, *Bull. Korean Chem. Soc.* **2002**, *23*, 1166-1168.

[8] B. Yang, J. Deng, H. Li, T. Yan, J. Zhang, D. Zhang, *iScience* **2021**, *24*, 102747.

[9] S. Zhang, L. Tang, J. Yu, W. Zhan, L. Wang, Y. Guo, Y. Guo, *ACS Appl. Mater. Interfaces* **2021**, *13*, 58605-58618.

[10] Q. Wang, C. Shen, J. Zhang, J. Wang, C. Liu, X. Wang, W. Ding, X. Guo, *Appl. catal., B Environ.* **2026**, *383*, 126105-126119.

[11] J. Kim, J.-C. Seo, W.-J. Jang, K. Lee, *ACS Sustainable Chem. Eng.* **2023**, *11*, 17415-17424.

[12] M. Bhattacharjee, T. Bhunia, A. Hossain, C. K. Mandal, P. K. Sinha, S. Ghosh, A. Bhaskaran, S. Roy, B. Show, M. M. Seikh, P. Bera, A. Gayen, *ACS Appl. Energy Mater.* **2025**, *8*, 10961-10973.

[13] Z. Wu, B. Yang, S. Miao, W. Liu, J. Xie, S. Lee, M. J. Pellin, D. Xiao, D. Su, D. Ma, *ACS Catal.* **2019**, *9*, 2693-2700.

[14] Q. Wang, W. Wang, M. Cao, S. Li, P. Wang, J. He, R. Li, X. Yan, *Appl. catal., B Environ.* **2022**, *317*, 121806.

[15] Y. Lu, L. Kang, D. Guo, Y. Zhao, Y. Zhao, S. Wang, X. Ma, *ACS Catal.* **2021**, *11*, 8749-8765.

[16] K. Y. Kim, J. H. Lee, H. Lee, W. Y. Noh, E. H. Kim, E. C. Ra, S. K. Kim, K. An, J. S. Lee, *ACS Catal.* **2021**, *11*, 11091-11102.
